# Influence of Template Size, Canonicalization, and Exclusivity for Retrosynthesis and Reaction Prediction Applications

**DOI:** 10.1021/acs.jcim.1c01192

**Published:** 2021-12-23

**Authors:** Esther Heid, Jiannan Liu, Andrea Aude, William H. Green

**Affiliations:** Department of Chemical Engineering, Massachusetts Institute of Technology, Cambridge, Massachusetts 02 139, United States

## Abstract

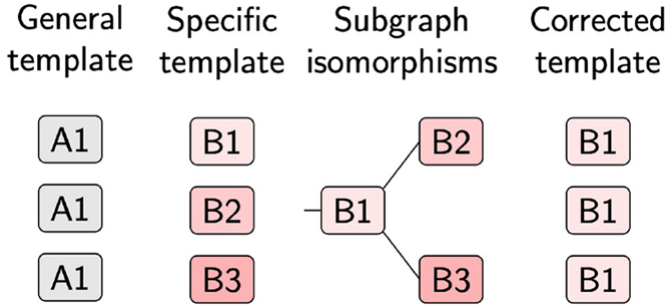

Heuristic and machine learning models for rank-ordering reaction templates comprise an important basis for computer-aided organic synthesis regarding both product prediction and retrosynthetic pathway planning. Their viability relies heavily on the quality and characteristics of the underlying template database. With the advent of automated reaction and template extraction software and consequently the creation of template databases too large for manual curation, a data-driven approach to assess and improve the quality of template sets is needed. We therefore systematically studied the influence of template generality, canonicalization, and exclusivity on the performance of different template ranking models. We find that duplicate and nonexclusive templates, i.e., templates which describe the same chemical transformation on identical or overlapping sets of molecules, decrease both the accuracy of the ranking algorithm and the applicability of the respective top-ranked templates significantly. To remedy the negative effects of nonexclusivity, we developed a general and computationally efficient framework to deduplicate and hierarchically correct templates. As a result, performance improved considerably for both heuristic and machine learning template ranking models, as well as multistep retrosynthetic planning models. The canonicalization and correction code is made freely available.

## Introduction

Retrosynthesis, i.e., the proposal of precursors for a desired product, and forward reaction prediction, i.e., the proposal of possible products given a set of reactants, are central topics of organic chemistry. With the surge of computer-aided reaction prediction approaches, numerous models for retrosynthesis based on heuristics^1^ and machine learning were developed, such as rule- or template-based models,^2−5^ transformer models adapted from natural language processing,^6−10^ or conditional graph logic networks.^11^ Despite the limitations of template-based approaches to generalize to new chemistries due to missing templates, their ability to fully specify precursors and to easily compare a proposed reaction to known reactions with similar transformations makes them a useful and valuable tool for synthesis planning software.^1^ Template-based models usually take a molecule as input and propose a ranked list of chemical transformations, often via flat multiclass classification. Since the training of multiclass models becomes more difficult with a larger number of classes,^12^ the performance of template-based models is influenced by the number of templates, and thus by their size, canonicalization, and exclusivity. Larger, more specific reaction templates include more atoms around the reaction center and only apply to a small number of molecules. Smaller, more general templates are applicable to more molecules and decrease the overall number of classes, potentially increasing model performance. However, they may lead to a large number of proposed precursors, some of which may not be chemically meaningful. Finding the optimal size of templates for an application is therefore an important and often ambiguous, undetermined problem. In contrast, poorly canonicalized templates (different templates describing the exact same transformation on the same set of molecules) or nonexclusive templates (different templates describing the same chemical transformation on overlapping sets of molecules) unnecessarily increase the number of templates, thus adding noise to the data and should be avoided if possible. However, data-driven approaches to retrosynthesis usually rely on the automated extraction of reaction templates from reaction databases, for example, via the open-source package RDChiral.^13^ Such template sets are, by nature, not as well curated and validated as manually crafted reaction rules. They can contain duplicate and nonexclusive templates and may also suffer from too large or too small template sizes. This necessitates the development of efficient and scalable canonicalization and correction routines.

Despite these challenges, the effects of template size, canonicalization, and exclusivity on model performance are hardly investigated. A recent study on the influence of the choice of data sets and template size found that smaller templates lead to a lower top-1 prediction accuracy, despite increasing model performance for multistep retrosynthesis and increasing applicability,^14^ which is counterintuitive from a machine learning point of view (where fewer classes should increase model accuracy). To the best of our knowledge, no methodical studies of the effects of template exclusivity and canonicalization have been published.

The aim of this study is therefore two-fold. First, we aim to characterize and quantify the effects of template size, canonicalization, and exclusivity on heuristic and machine learning template-ranking algorithms for retrosynthesis and forward prediction applications. Second, we aim to establish new canonicalization and hierarchical correction algorithms to remedy the two most important issues identified: duplicate and nonexclusive templates. The resulting code is freely available on Github.^15,16^

## Methods

### Data Sets

This study relies on two data sets, which were both prepared from reactions extracted from the United States Patent and Trademark Office (USPTO) by Lowe and coworkers.^17,18^ First, a subset of 50,000 pharmaceutically relevant reactions, curated by Schneider et al.^19^ and employed in a variety of previous studies,^1,5^ was used as provided by ref (1) and the preprocessing steps described therein. Second, a set of 480,000 USPTO reactions collected for forward reaction prediction^20^ and employed in various studies^21,22^ was utilized. This data set was further processed by removing molecules from the reaction that did not contribute a heavy atom to the product (code from ref (5)), as well as removing reactions with more than one product molecule.

For both data sets, templates in the retrosynthetic direction were extracted via the RDChiral Python package^13^ with default settings (radius 1 and inclusion of certain special groups), as well as at radius 3, 2, 1, and 0 without any special groups after modifying the code slightly.^23^ Leaving groups were included in all extracted templates. Only reactions yielding a valid SMARTS string as template, as well as reproducing the reactants after application to the products, were kept, yielding 48,531 reactions in the smaller data set, termed USPTO-50k throughout the remainder of this article, as well as 461,541 reactions for the larger data set, termed USPTO-460k. Templates for forward reaction prediction were obtained by simply reversing the direction of change of the retro templates. Since RDChiral is designed to extract templates in the retrosynthetic direction and imposes certain chirality restrictions which are only meaningful in that direction, reversing the templates may make them less general than necessary. As this study is primarily concerned with the relative effects of canonicalization and exclusivity, the forward (reversed retrotemplates) were not sanitized further.

### Model Details

Each data set was split into training, validation, and test reactions via a random 80%/10%/10% split. Three different models were then trained to recommend templates which reproduce the observed reaction at high ranks. Either the product molecule was employed as input for the task of predicting retrosynthetic disconnections or the reactant molecule(s) for the task of predicting the reaction outcome in the forward direction. First, the heuristic template ranking procedure described in ref (1) was utilized as a baseline (referred to as “Sim”), which ranks precedent reactions based on Tanimoto similarities of Morgan fingerprints,^24^ as implemented in RDKit.^25^ Second, a neural network, similar to the baseline model in ref (5) was trained on Morgan fingerprint bit vectors of the products or reactants (referred to as “ML-fixed”). Lastly, a graph convolutional neural net, Chemprop,^26^ was employed to encode a molecule and predict a template class (referred to as “ML-learned”). This model does not rely on precomputed fingerprints, but creates its own, task-specific learned embedding of the molecule via a directed message passing neural net, which is then followed by a standard feed forward neural net. Further details on each model and their hyperparameters are given in the Supporting Information.

For each model, the number of applicable templates in the top-*N* ranked templates, as well as the top-*N* accuracy, were calculated. A recent study found that top-*N* accuracy should not be used as a single metric to evaluate the fitness of a template ranking model and should instead be accompanied by a measure of template applicability, as well as the ability of the model to produce viable synthetic routes for target products.^14^ Therefore, the number of applicable templates recommended by each model was analyzed in addition to top-*N* accuracy, i.e., the number of templates that produce any valid precursor or product. In our top-*N* accuracy metrics, two different definitions of success were considered. Most machine learning models define success as recommending the exact template associated with a test reaction in the data set. Less commonly, success can be defined via recovering the actual molecules in the test reaction (precursors or products, depending on the direction of the template) after application of the recommended template. If all templates are mutually exclusive, both metrics of success yield the exact same results, which is highly desirable but often not achieved in practice, as shown later in this article. Below, metrics based on the “exact template” criterion are labeled T and those based on “exact precursors” or “exact product” are labeled P.

We furthermore retrained the policy neural network of the Monte Carlo tree search retrosynthesis planner AiZynthFinder^27^ with the reactions and templates from the current study to evaluate the ability of different template sets and their corresponding template recommendation models to create synthetic routes. Default parameters were used as described in ref (27), where we only retrained and utilized the policy model, not the filter model. The processed template and model files are available on Github.^15^

### Canonicalization of Templates

Canonicalization of a template is a process that generates a unique string representation of the chemical transformation, which typically consists of a pair of SMARTS connected by the atom mapping. Due to the uniqueness of the canonical form, this process is crucial in template indexing and deduplication. The problem is a generalization of canonicalization of SMILES/SMARTS and mathematically equivalent to computing the canonical form of a graph.^28,29^ The problem is NP, but its exact time complexity is still unknown.^30−33^ In this section, instead of discussing the exact solutions, we attempt to present a practical, open-source approach to the template canonicalization problem and discuss its limitations and alternative solutions. We furthermore note that we only attempt to canonicalize templates as output by RDChiral, which significantly simplifies the task, since RDChiral only produces simple SMARTS patterns without negations, recursions, or wildcards. For comparisons between more complex SMARTS patterns, we refer the interested reader to the seminal work of Rarey and coworkers on detecting equality and hierarchy between SMARTS patterns.^34,35^

Compared to the SMILES canonicalization problem, the template canonicalization problem has two major differences: (1) A template comprises reactant and product SMARTS, in other words, two graphs instead of one. (2) The two graphs are further connected through the atom mapping. Hence, it is natural to merge the two graphs into one condensed graph^36^ if two nodes have the same atom mapping number. Here, we note that standardizing the atom mapping is also necessary to produce a unique representation.

The key to the canonicalization problem is the node ranking algorithm, which computes the canonical rank of each node or atomic query in SMARTS for this particular problem. We adopted the Weisfeiler–Lehman refinement^37^ as our ranking method. It iteratively relabels each node *v* ∈ *V* on the graph *G*(*V*, *E*) using the features from the node *v* itself and its neighbors , until the partition of all the labels becomes stable or the cycles are repeated at least |*V*| times, where |*V*| is the number of nodes. We chose node degree and canonical atomic SMARTS without the atom mapping number as the node features and bond SMARTS as the edge features, both with chirality included. Since the condensed graph was employed, the node features from the reactant and the product graphs were simply concatenated if they shared the same atom mapping number. In addition, a tie-breaking tag was also included. A detailed explanation of the canonicalization process is given in the Supporting Information.

A major limitation in our algorithm is the ranking method itself, which is equivalent to the 1-dimensional Weisfeiler-Lehman refinement, and cannot distinguish certain highly symmetric graphs.^33,38,39^ As a consequence, our canonicalization algorithm may not produce a unique representation for some templates. Despite this limitation, it does not affect this study because a) the number of nonunique representations is very small and b) the template correction algorithm applies a subgraph isomorphism test to compute the hierarchy. The frequency of nonunique representations can be further reduced by including more heuristic invariants,^33^ e.g. as the ones used in RDKit.^28^ If it is required to guarantee the uniqueness of the template string, the Weisfeiler-Lehman refinement needs to be replaced by a more general graph canonicalization algorithm with the price of potentially higher time complexity, e.g. the nauty algorithm.^40^

In the end, we note that our current implementation of the canonicalization algorithm in the RDChiral C++ package^16^ only supports the **And** operator in atomic SMARTS query, which is sufficient to canonicalize template patterns output by RDChiral. However, it should be possible to extend our code to support other SMARTS queries or use a more general SMARTS comparison algorithm such as SMARTScompare.^34,35^ We furthermore note that the C++ version^16^ of RDChiral is only used for template canonicalization; in all other parts of the workflow, we used a modified RDChiral Python package.^23^

### Hierarchical Correction of Templates

**Figure 1:**
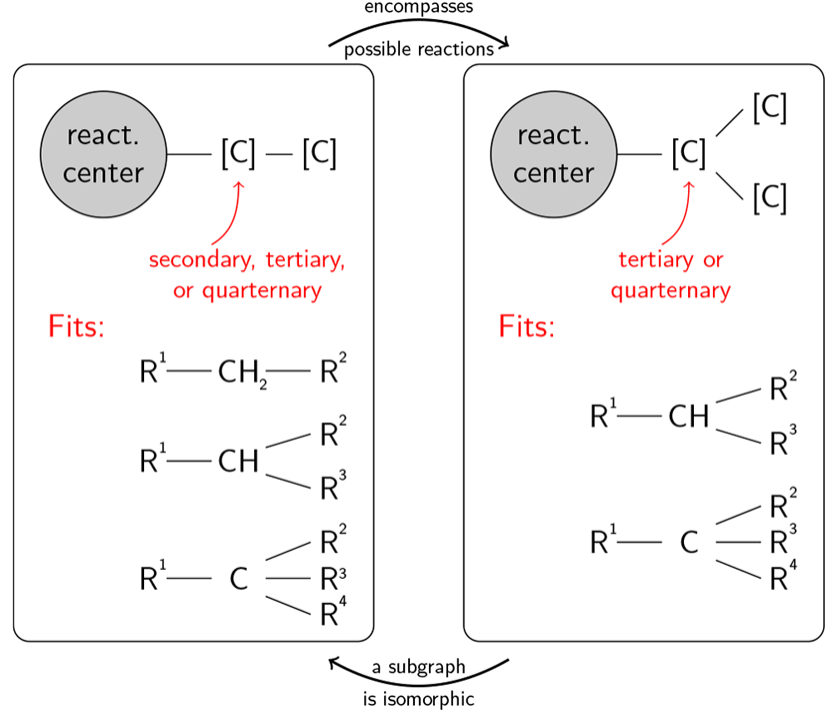
Example of a template (right) with a subgraph that is isomoporphic to another template (left). Within the correction algorithm, only the left template is kept, since it encompasses all possible reactions of the right template.

In a manual examination of the extracted templates, we found that nonexclusivity of templates can stem from multiple sources. With the default template extraction parameters of RDChiral (radius 1, with special groups), for example, templates are extracted around the reactive center up to one bond away, and further atoms are attached if they match a set of expert-crafted special groups. Multiple reactions might thus yield templates with the same chemical transformation within the same context up to radius 1 but might include different, or no, special groups. A number of examples can be found in the Supporting Information, Figure S1. If a set of templates includes one instance without special groups and one with a special group, the templates are not mutually exclusive anymore; i.e., the template without the special group is applicable to all other reactions with the same template at radius 1. Templates at large radii, especially at radius 2 and 3, can furthermore be nonexclusive if some of the reactions they were extracted from contained a branched side chain. An example is given in Figure 1, where the left abstracted structure fits the same and more molecules than the right abstracted structure. Therefore, the template on the right possesses a subgraph which is isomorphic to the template on the left, and only the more general template should be kept. Further examples of this behavior are given in the Supporting Information, Figure S2. If templates describe the same transformation on an overlapping set of molecules, as in Figure 1, mutual exclusivity can be enforced by keeping only the most general template. To identify the template encompassing all relevant reactions, subgraph isomorphisms need to be calculated between pairs of templates. Due to the computational inefficiency inherent to subgraph isomophism evaluations, an exhaustive comparison of all possible pairs of templates in a set is unfeasible; instead, we developed a hierarchical alternative which narrows down the number of necessary subgraph searches. Toward this aim, we utilized RDKit to detect subset relations between templates output by RDChiral, which only comprise simple SMARTS patterns. For a comparison of more complex SMARTS patterns, packages such as SMARTScompare^34,35^ may be employed. We further note that an exhaustive comparison of all templates in a set can result in unwanted matches for templates with different reaction centers (example shown in the Supporting Information), which is circumvented by our hierarchical approach as explained in the following.

**Figure 2:**
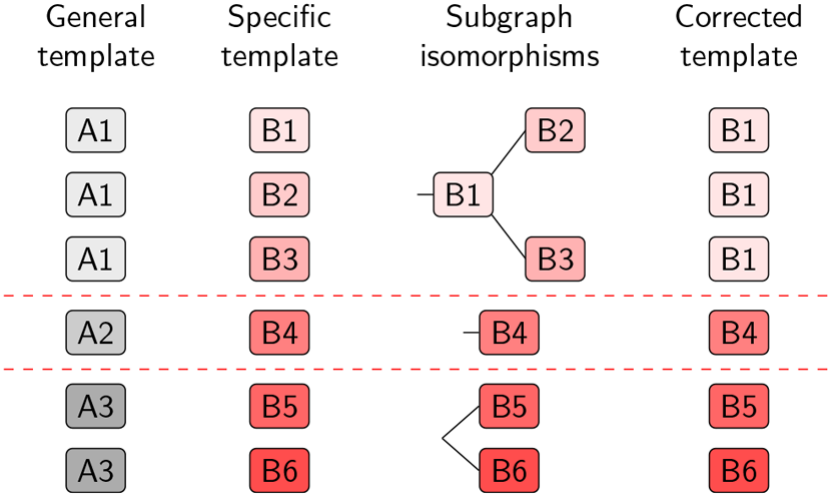
Schematic depiction of the hierarchical correction algorithm. Templates are clustered using a general template representation (for example, templates B1–B3), and subgraph isomorphisms are computed within each cluster to identify the most general, exclusive patterns (for example, B1). Templates with a more general parent within a cluster are then replaced.

Figure 2 schematically depicts how the hierarchical correction algorithm detects and eliminates nonexclusive templates. Templates at the desired level of specificity (with SMARTS strings B1–B6, red shades), for example, at radius 1, are clustered according to their respective templates at a lower level of specificity (with SMARTS strings A1, A2, and A3, gray shades), for example, at radius 0. The general templates (gray) are only used to group the more specific templates and thus lower the number of subgraph isomorphism evaluations but are not taken into account beyond that. The grouping also makes a comparison of atom map number between the matches obsolete because it enforces that the same chemical transformation is encoded in each template pair. Furthermore, templates extracted by RDChiral follow a specific syntax, where more strict SMARTS strings are assigned to atoms in the reaction center and more general SMARTS strings to all other atoms,^13^ which ensures that the correct substructures of a pattern are matched to each other without inspecting atom map numbers. For templates extracted with different software packages, we recommend including a matching of the atom map number in each subgraph isomorphism search and clustering according to the minimal, most general template/reaction center. Within each cluster, subgraph isomorphisms are calculated between pairs of templates, leading to a tree structure of subgraph isomorphism relationships as depicted in the third column in Figure 2. For example, for templates in the A1 group, B1 is more general than both B2 and B3 and encompasses both of them. Thus, only B1 is kept. The second group, A2, contains only a single template, and is kept as is. The third group, A3, contains two templates that do not encompass each other, i.e., are unique, so both B5 and B6 are kept. In practice, a combination of these cases can occur, leading to more complex trees. To further lower the computational load, subgraph comparisons do not need to be exhaustively calculated between all templates in a group. Instead, the trees are built iteratively, where a new template is only compared to the current most general template(s) but not to templates already labeled as nonexclusive or duplicates. Further details of the hierarchical correction algorithm, as well as a pseudocode representation, are given in the Supporting Information. We note that an analogous, pairwise comparison algorithm can furthermore be easily implemented via other SMARTS pattern comparison algorithms. The hierarchical correction code used in this study is available on Github.^15^

In the following, “corrected templates” indicates that template sets were pruned according to the hierarchical correction algorithm. Radius 1 templates were corrected first with clustering at radius 0; then, default and radius 2 templates were corrected with clustering at corrected radius 1. Finally, radius 3 templates were corrected with clustering at corrected radius 2. Since the templates at radius 0 cannot be corrected with the developed algorithm, proper canonicalization is especially important at radius 0; otherwise, errors propagate up the hierarchical scheme, where the clustering does not correctly group together all relevant templates.

One may argue that the exclusivity issues, at least for default templates (at radius 1 with special groups), could be resolved by omitting some or all special groups. However, the hierarchical correction features a useful side effect for this class of templates. Namely, special groups which are not important in a specific chemical transformation according to the database, i.e., which are not specified in all reactions, are removed automatically. On the other hand, special groups that are important (one special group occurs in all reactions or different special groups occur exclusively) or special groups in rare templates (with only one reaction precedent) are kept. Thus, the inclusion of special groups is handled automatically based on the context around the reaction center as extracted from a set of reactions and does not depend on an *a priori* choice, such as to remove selected special groups to allow for more general templates.

Furthermore, an alternative path to enforce exclusivity was explored, namely, to specify the number of hydrogen atoms for each template atom, which solves some of the issues encountered at radius 2 and 3 due to branched versus linear side chains. However, this decreased model performance and template applicability considerably (data shown in the Supporting Information) and did not resolve a number of nonexclusivity issues, for example, due to special groups, so this approach was not pursued further.

## Results and Discussion

In order to assess the effects of duplicate and nonexclusive templates on the performance of template ranking algorithms, it is necessary to first create a clean set of templates. To clear out duplicates, the newly developed iterative canonicalization process for SMARTS templates extracted with RDChiral was applied. To filter out nonexclusive templates, our novel hierarchical correction scheme was utilized to arrive at exclusive template sets.

We now explore the effects of canonicalization and correction on the number and popularity of unique templates, as well as the performance of template ranking algorithms.

### Popularity and Number of Unique Templates

**Figure 3:**
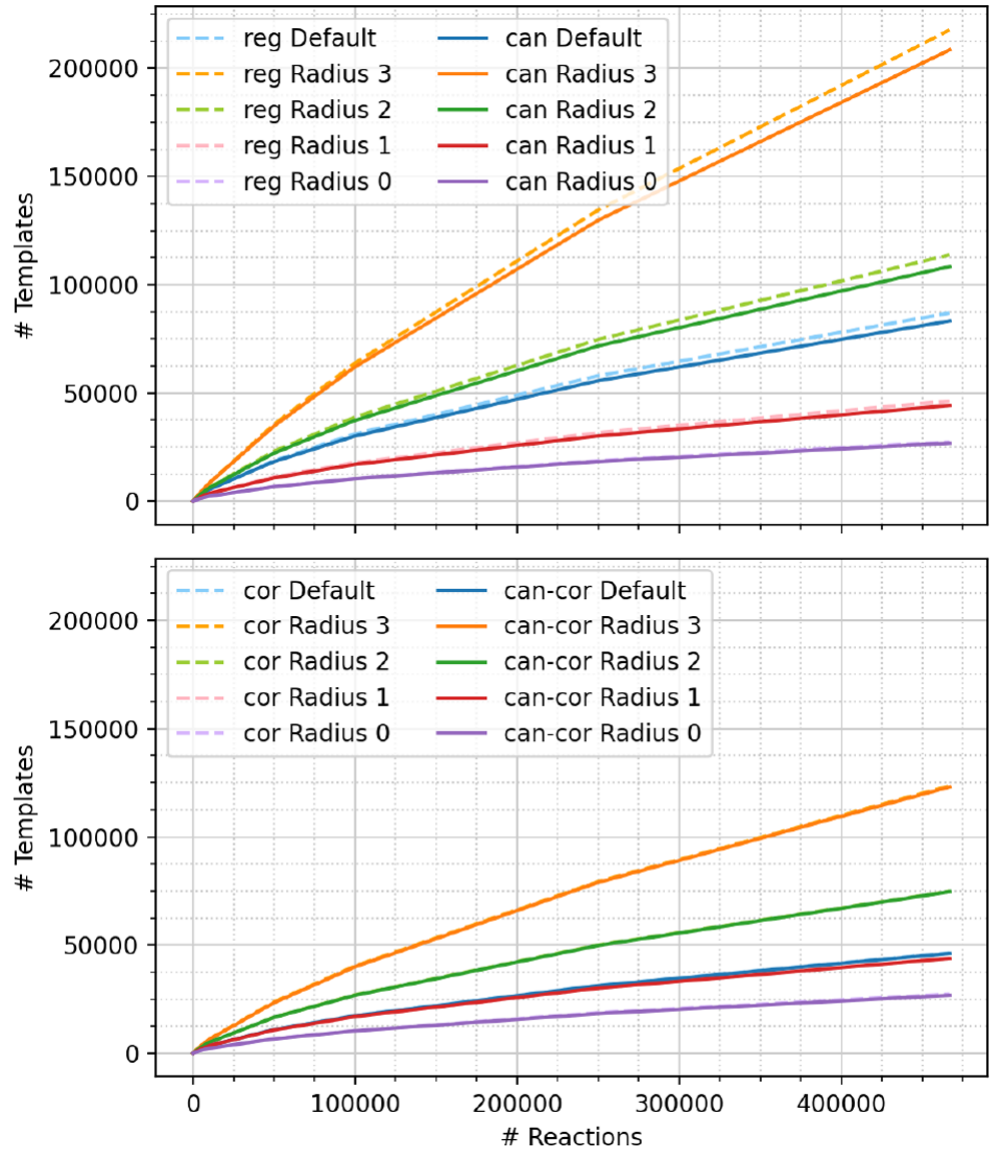
Comparison of the number of templates (top, regular and canonical; bottom, hierarchically corrected and canonical + corrected) per number of reactions for subsets of the UPSTO-460k data set. The “cor” and “can-cor” curves overlap on this scale.

**Figure 4:**
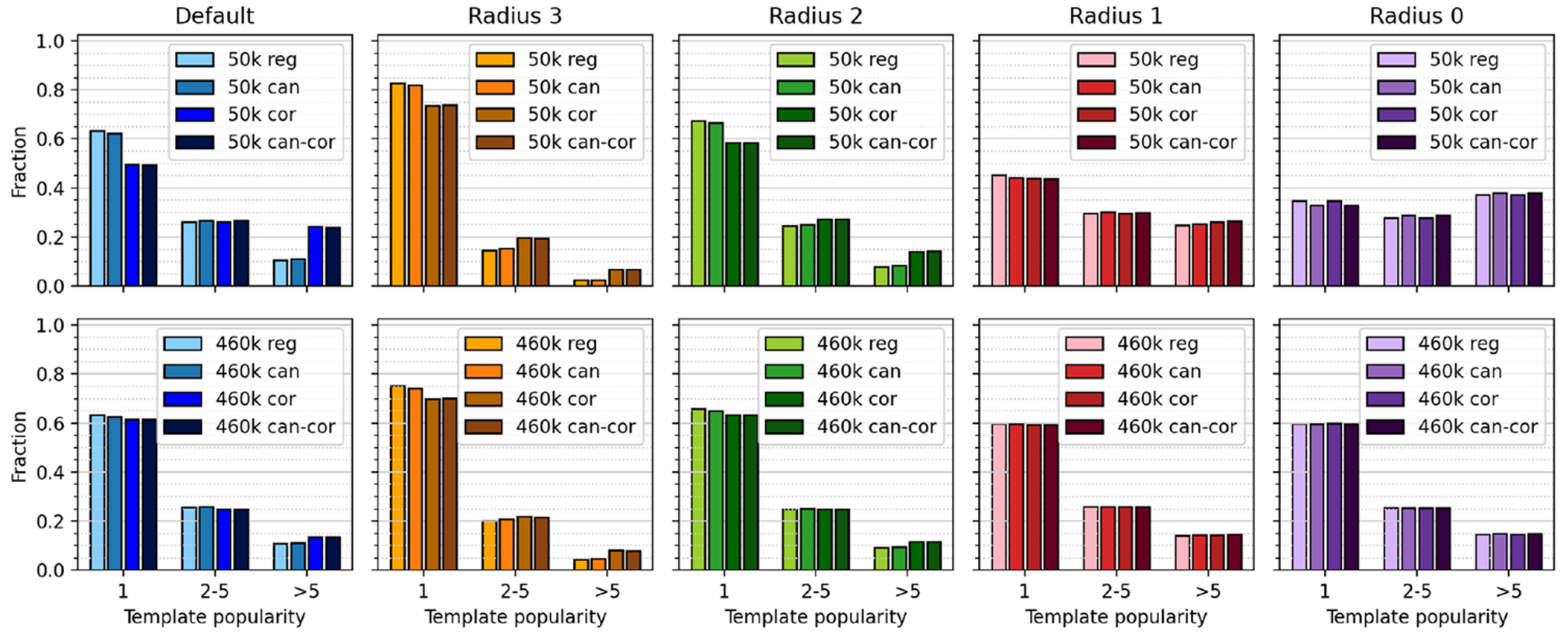
Histogram of number of reactions associated with each template for different template sizes and canonicalization/correction schemes for USPTO-50k (top) and USPTO-460k (bottom).

Figure 3 depicts the number of templates as a function of data set size, calculated on the USPTO-460k data set and subsets thereof. More specific templates extracted at larger radii naturally yield a larger number of extracted templates for a given data set. For the largest templates studied (radius 3, with no correction, no canonicalization, labeled “reg Radius 3”), about every second new reaction leads to a new template, whereas only every twentieth reaction actually encodes a new transformation, visible from the number of templates at radius 0. Since the accuracy of a machine learning template recommendation scheme usually suffers from a large number of templates (corresponding to a large number of classes in the multiclass classification task), it is desirable to keep the number of templates as low as possible, without sacrificing chemical plausibility of the recommended reactions. Additionally, it is desirable to keep the number of templates associated with only one or a few reactions as low as possible, since the ability of machine learning models to recommend such rare templates is usually low.^5^Figure 4 depicts the popularity of the extracted templates from USPTO-50k and USPTO-460k for different template sizes. More general, smaller templates lower the fraction of rare templates occurring only once in the whole data set, especially for the smaller USPTO-50k data set.

Figures 3 and 4 furthermore depict the influence of hierarchical template correction (labeled “cor”) and canonicalization (labeled “can”). The correction procedure lowers the number of unique templates as well as lowers the number of rare templates. For default RDChiral templates (radius 1 + special groups), the correction scheme removes most of the special groups. Without the correction schemes, the extracted templates contain many duplicates (same transformation, but encoded as different SMARTS string), so that canonicalization decreases the number of unique template strings (top panel in Figure 3). After hierarchical correction, canonicalization does not change the number of templates considerably for radii larger than 0; i.e., there is nearly no difference between corrected, “cor”, and canonical corrected “can-cor” templates. Since duplicates at radius 0 can propagate up the hierarchical correction scheme, ideally canonicalization and correction are combined (“can-cor”).

From the absolute number of templates, and the number of reactions associated with each template, we can thus conclude that the hierarchical correction scheme not only efficiently reduces the overall number of templates but also the fraction of templates associated with only a single reaction. If a hierarchical correction is not possible or wanted, canonicalizing templates is an alternative, cheaper possibility to decrease the overall number of templates, but to a much smaller extent.

### Influence of Template Characteristics on Model Performance

In the following, we examine the model performance of the similarity, ML-fixed, and ML-learned model across different template sizes, correction, and canonicalization schemes. The ranking ability of a model was evaluated via top-*N* accuracy, where the fraction of test reactions correctly recovered in the top-*N* ranked suggestions is measured. Two different success criteria were employed, namely, recommending either (a) the exact template extracted for a test reaction or (b) the exact molecules in the test reaction produced by template application. For a set of exclusive templates, these definitions lead to identical results, but a discrepancy arises for nonexclusive templates, where different templates lead to the same outcome, thus rated as success when comparing outcomes but as failure when comparing templates. We use this discrepancy as a measure of nonexclusivity in the following.

**Figure 5:**
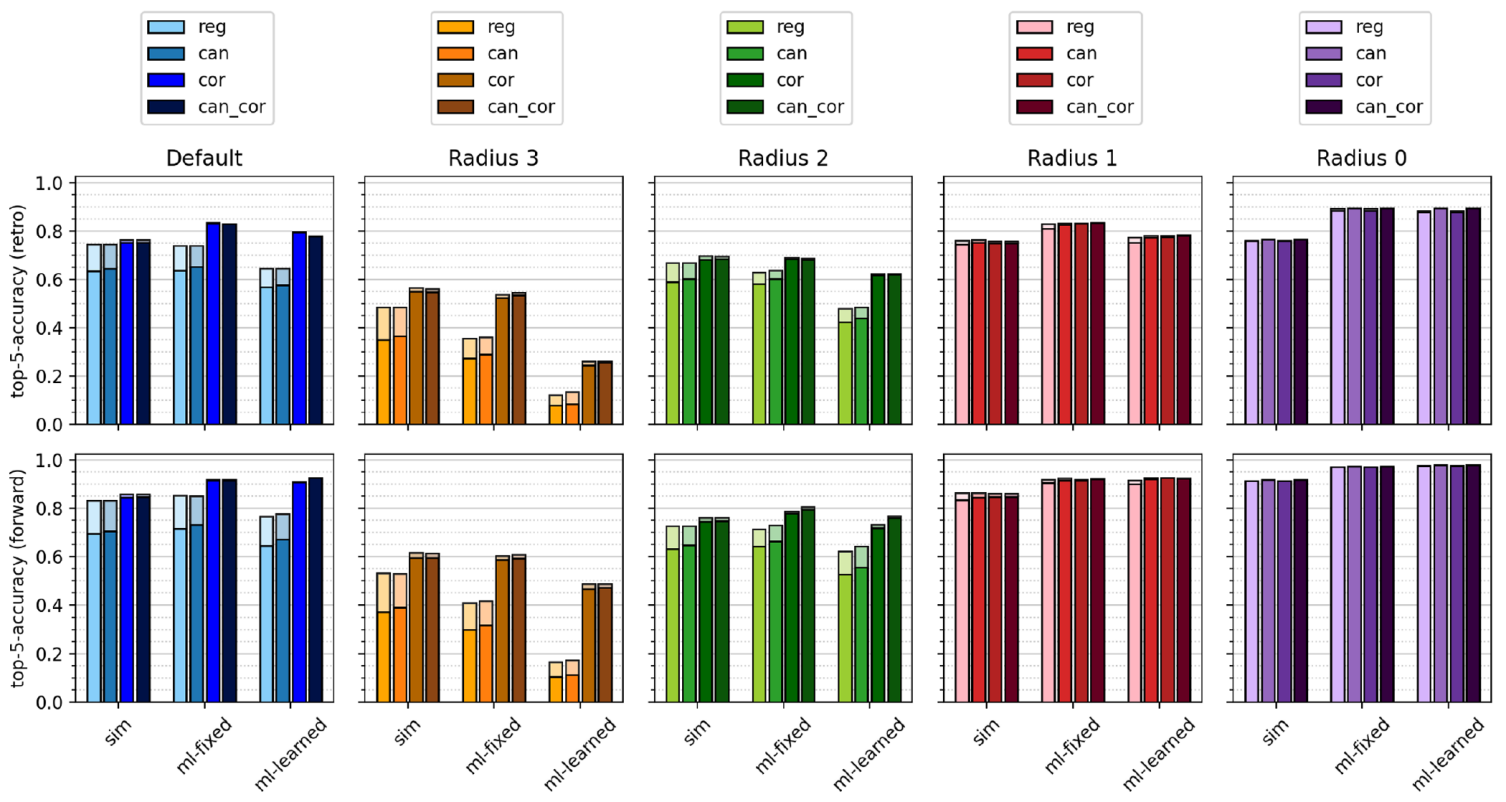
Top-5 accuracies of proposed retrosynthetic disconnections (top) and forward predictions (bottom) for the USPTO-50k data set using the “sim”, “ml-fixed”, and “ml-learned” models. The darker shade in a bar corresponds to evaluation via comparing templates and the lighter shade to comparing precursors or products. Each set of four bars shows the effects of canonicalizing (“can”) or hierarchically correcting (“cor”) the regular uncorrected templates (“reg”) or both (“can-cor”).

**Figure 6:**
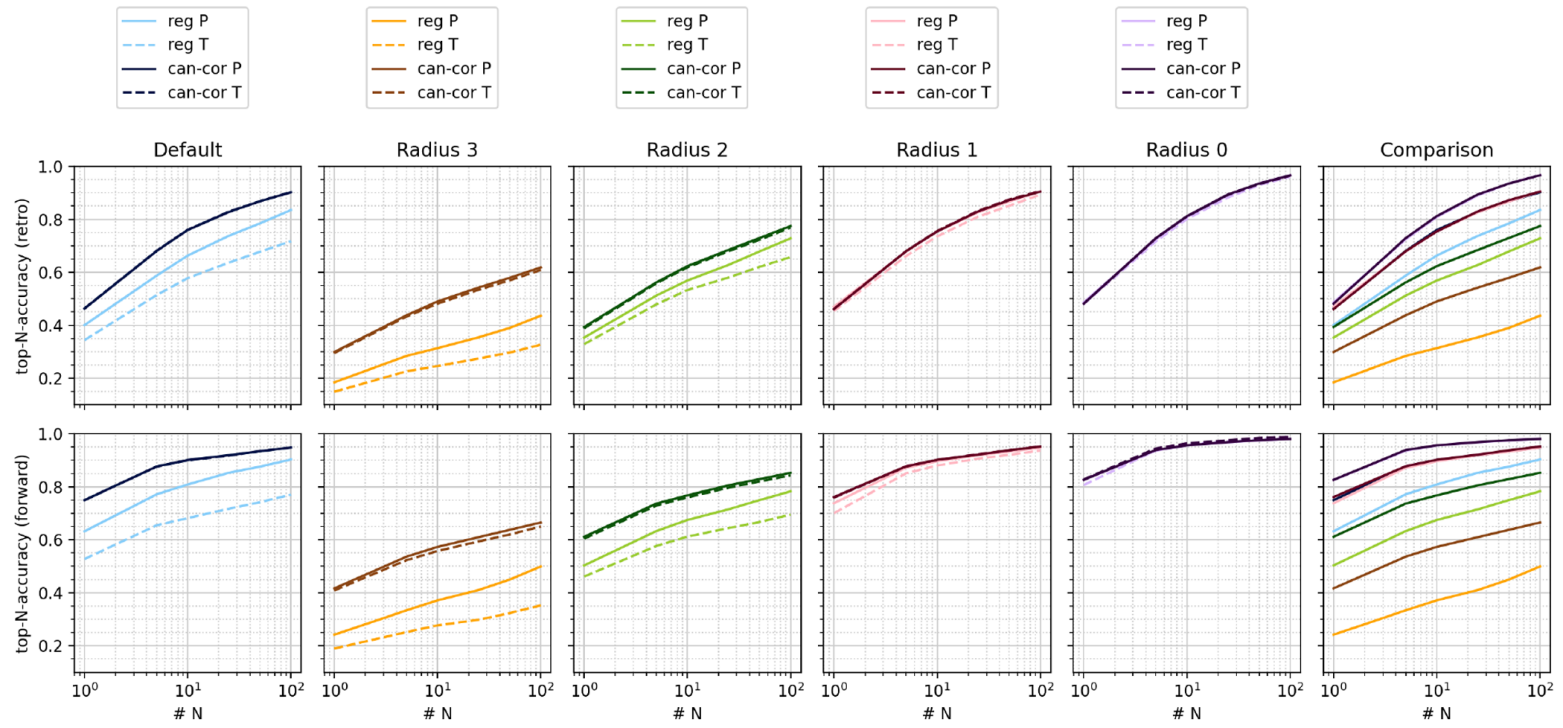
Dependence of top-*N* accuracies of proposed retrosynthetic disconnections (top) and forward predictions (bottom) on the template scheme for the USPTO-50k data set, ML-fixed model. Here, “reg” corresponds to uncorrected and “can-cor” to canonical and corrected templates. P means evaluated by precursors or products (continuous line); T means evaluated by template match (dashed line).

Figure 5 depicts the top-5 accuracies across the different models, template sizes, correction/canonicalization, and success criteria. The bars with darker shade correspond to success via identical templates and the bars with lighter shade to success via reaction outcome after template application, so that the visible lighter area quantifies the effects of nonexclusive templates. For all models and correction/canonicalization schemes, smaller template sizes lead to higher model performance. Correction/canonicalization increases model performance for both evaluation criteria but especially for success defined via identical templates, where the hierarchical correction remedies the effects of nonexclusive templates and boosts model performance across all models for default, radius 2, and radius 3 templates. We furthermore note that the ML-fixed model performs equally well or better than the ML-learned model across all systems and outperforms the similarity model for some systems only after template correction. The ML-learned model performs especially poorly for large sets of templates, for example, uncorrected templates at radius 3 or 2 or at radius 1 with special groups and thus classification tasks with a large number of classes. The ML-learned model is faced with a more difficult learning objective than the ML-fixed model since it learns both the molecular embedding from the molecular graph and the template class from the embedding, as opposed to the ML-fixed model, which only learns the latter. Both the larger number of parameters and the additional task make the ML-learned model more prone to overfitting, which requires more data to find a meaningful relation between a template and a molecular graph. For template sets with a large number of classes, most templates are only associated with a single reaction, so that the data are insufficient to learn a meaningful molecular embedding (see SI for further details). The ML-learned model therefore profits greatly from template correction. The positive effects of template correction are larger for both machine learning models than for the heuristic model, which is to be expected from the reduction of classes and thus a more effective training of classification models. Top-*N* accuracies for *N* = 1 and *N* = 50 are shown in the Supporting Information, as well as similar figures for the USPTO-460k data set. Figure 6 depicts top-*N* accuracies for different values of *N* for the uncorrected templates (“reg”) and canonical + corrected templates (“can-cor”) across different template sizes for the ML-fixed model. For regular templates, the discrepancy between success via templates (dashed) and success via outcomes (continuous) is very large for default, radius 2, and radius 3 templates and hampers model performance. Canonicalizing and correcting the templates hierarchically resolves the discrepancies, which boosts model performance considerably. Similar trends were identified for the USPTO-460k data set (Supporting Information), although the performance increase is not as large.

**Figure 7:**
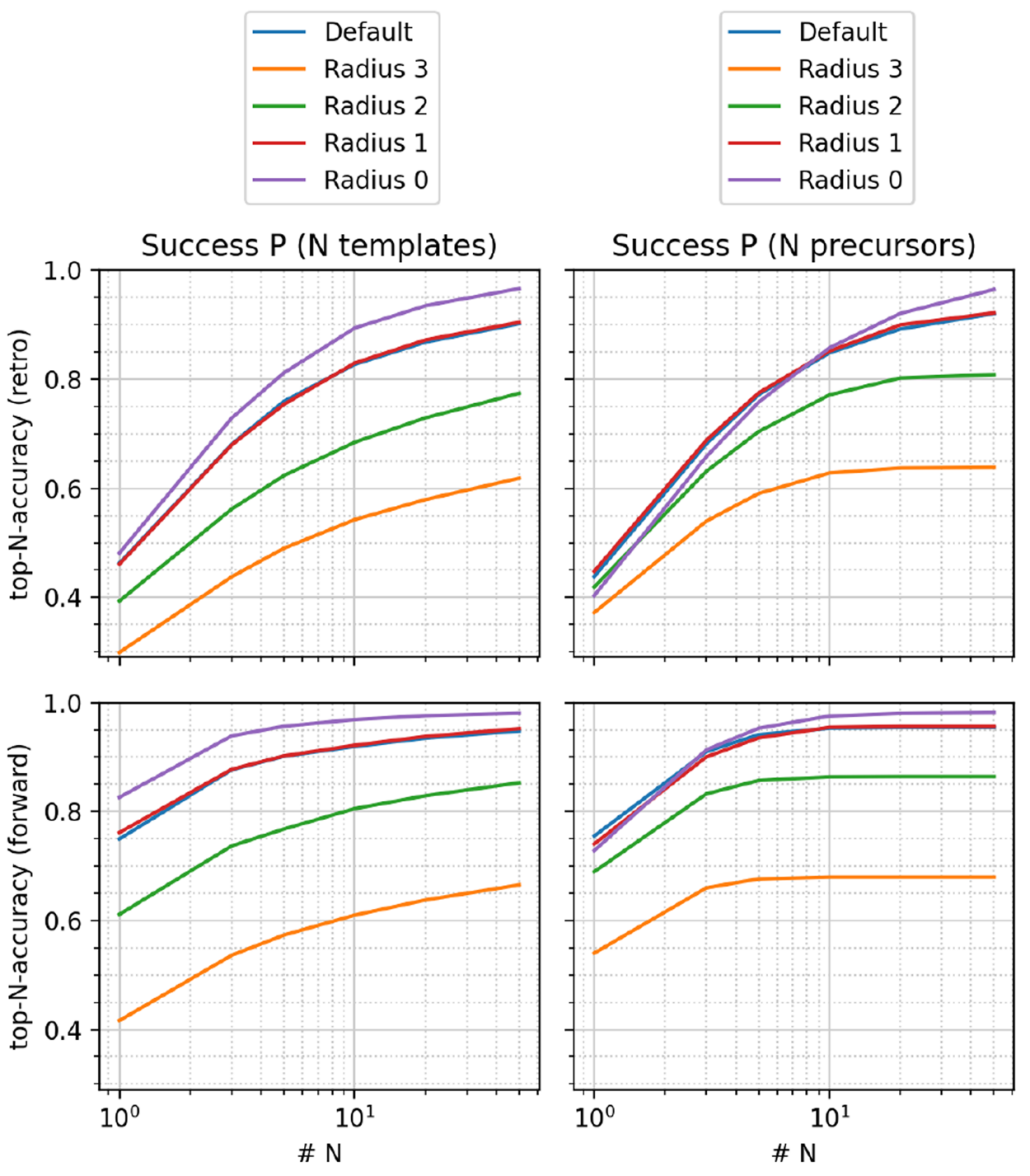
Top-*N* accuracies of proposed retrosynthetic disconnections (top) and forward predictions (bottom) for the USPTO-50k data set, canonical-corrected templates, ranking via the ML-fixed model. Left: ranking via the number of templates. Right: ranking via the number of precursors.

Canonicalizing and correcting templates thus offers a simple and accessible route toward smaller, more efficient template sets. But which template size is ideal? From Figure 5, one may conclude that the smaller the template is, the higher the model performance is. However, this evaluation does not take into account the number of produced precursors. For large radii, the application of a template usually produces a single (or no) reaction outcome. In contrast, small templates tend to produce a large number of reaction outcomes. For example, if the top-ranked template produces three precursors, and one of them coincides with the true reaction outcome, the three suggestions are not inherently ranked and should count toward top-1 accuracy only in 33.3% percent of cases. Figure 7 compares the observed top-*N* accuracies after accounting for this effect, namely, averaging the ranks over the produced outcomes (right) instead of simply checking whether the correct outcome was produced at a specified rank (left). In that case, templates at radius 0 become undesirable for both forward and retro models, as their top-1 accuracy drops considerably below default and radius 1 templates. A similar behavior was found for USPTO-460k (Supporting Information). We therefore recommend the use of default templates together with the canonicalization and correction schemes developed in this study. Templates at radius 1 lead to an acceptable performance, too, but we do not recommend them as unequivocally due to the following reasons. The hierarchical correction of default templates enables an automated pruning of special groups without an *a priori*, expert-guided selection. Since some special groups in RDChiral are necessary for a correct processing of stereochemistry,^13^ simply removing all special groups to arrive at radius 1 templates may have undesired side effects. The correction scheme only removes special groups which are not necessary or unique, i.e., correspond to a transformation that can be described fully and without information loss by another, more general template. It thus constitutes a more general, data-driven approach to select important special groups, while keeping the set of templates as small as possible.

**Figure 8:**
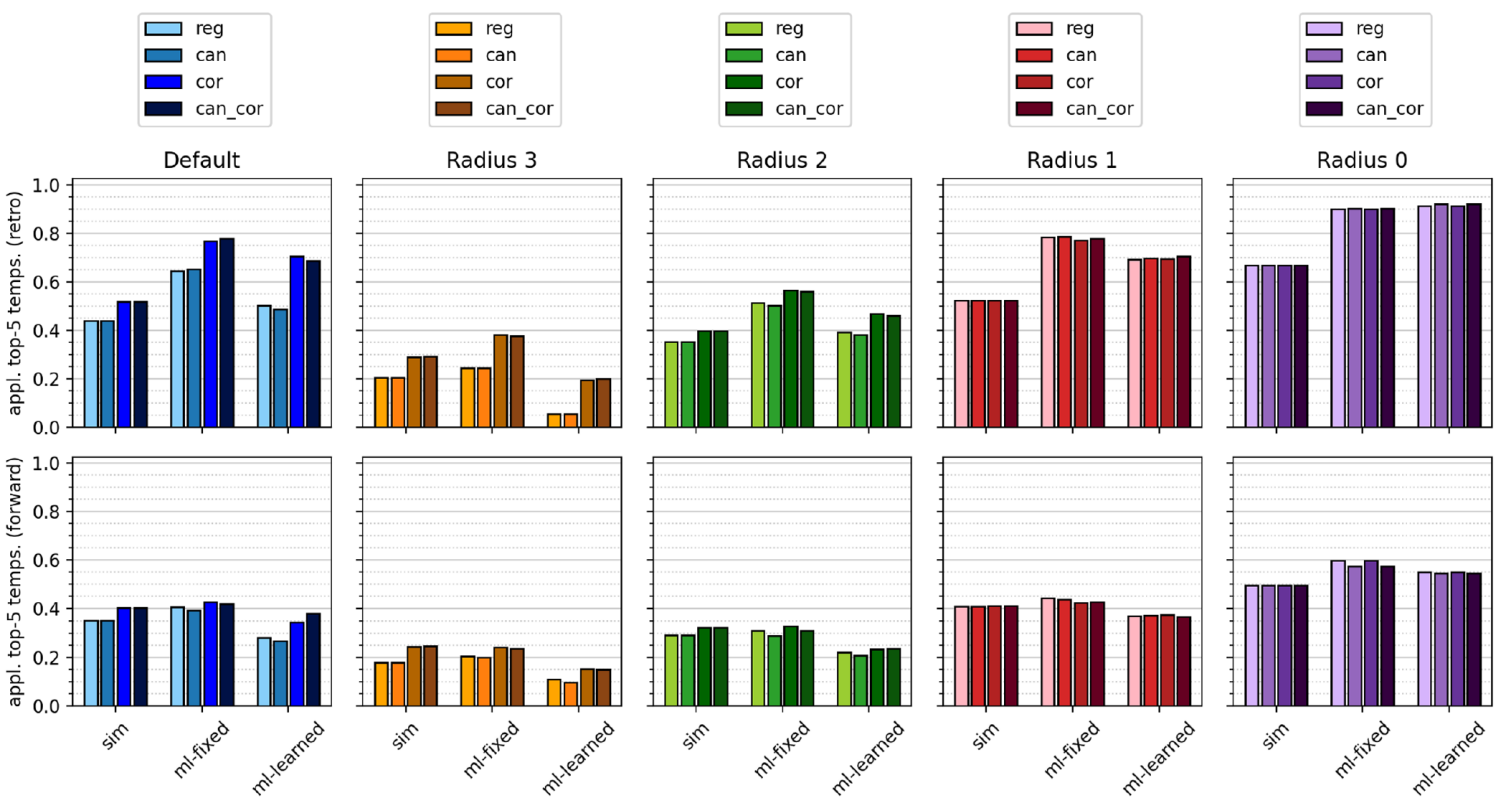
Fraction of applicable templates of the five highest ranked templates of proposed retrosynthetic disconnections (top) and forward predictions (bottom) for the USPTO-50k data set using the “sim”, “ml-fixed”, and “ml-learned” models. Each set of four bars shows the effects of canonicalizing (“can”) or hierarchically corecting (“cor”) the regular uncorrected templates (“reg”) or both (“can-cor”).

Interestingly, canonicalization and correction do not exclusively boost the performance of machine learning ranking models, which was expected since their training loss relies on the assumption that classes are mutually exclusive. Rather, the performance of the heuristic ranking model increases too, despite the fact that the heuristic ranking algorithm is not affected by template characteristics at all. This is a direct effect of the increased applicability of canonical and corrected templates. Figure 8 depicts the fraction of the five highest ranked templates that are applicable to the input molecule, i.e., produces one or more reaction outcomes. Applicabilities for the top-1 and top-50 templates, as well as for the USPTO-460k data set, are shown in the Supporting Information. For both forward and retro models, the hierarchical correction scheme significantly increases the applicability of default, radius 2, and radius 3 templates. We note that the ML-fixed algorithm performs best in ranking applicable templates highest, across all template sizes.

### Comparison to Other Template Ranking Models

As shown in Table 1, without any optimization of the model, the performance boost due to correction (retrosynthesis direction, default templates) leads our simple machine learning model to outperform the template-based models NeuralSym^2^ and Retrosim^1^ in top-*N* accuracy for all *N*, as well as GLN^11^ for *N* ≥ 5. Compared to semitemplate based models, this work outperforms the models G2Gs^43^ and RetroXPert^44^ for *N* ≥ 3, as well as GraphRetro^41^ for *N* ≥ 5. The template-free models SCROP^45^ and LV-Transformer^46^ are outperformed for all *N*, as well as DualTF^47^ and RetroPrime^48^ for *N* ≥ 5 (data from refs (41 and 42), USPTO-50k). Only the current state-of-the-art models DualTB,^47^ MEGAN,^42^ and AT (100x)^10^ yield better performances. Without correction, the employed model outperforms none of the mentioned models, highlighting the power and influence of the developed template correction algorithm. A full table for top-*N* accuracies and applicabilities for all investigated systems is given in the Supporting Information.

**Table 1:** Comparison of Performance of ML-Fixed Model (Evaluation via Template Identity) Trained on Default Templates with and without Correction and Canonicalization to Performance of Selected Retrosynthesis Models in Literature^a^

	Top-*N* accuracy (%)			
Model	*N* = 1	*N* = 3	*N* = 5	*N* = 10
This work, reg	34.4	51.4	57.8	63.7
This work, can-cor	46.4	68.2	76.0	82.9
NeuralSym^2^	44.4	65.3	72.4	78.9
G2Gs^43^	48.9	67.6	72.5	75.5
RetroXPert^44^	50.4	61.1	62.3	63.4
SCROP^45^	43.7	60.0	65.2	68.7
LV-Transformer^46^	40.5	65.1	72.8	79.4
DualTF^47^	53.6	70.7	74.6	77.0
Retrosim^1^	37.3	54.7	63.3	74.1
GLN^11^	52.5	69.0	75.6	83.7
DualTB^47^	55.2	74.6	80.5	86.9
GraphRetro^41^	53.7	68.3	72.2	75.5
MEGAN^42^	48.1	70.7	78.4	86.1
RetroPrime^48^	51.4	70.8	74.0	76.1
AT (100x)^10^	53.2		80.5	85.2

a Data from refs (41 and 42), USPTO-50k.

### Influence of Template Characteristics on Retrosynthesis Performance

To showcase the influence of deduplication and exclusivity of template sets on computer-aided retrosynthesis platforms, we retrained the policy network of AiZynthFinder^14,27^ using the regular and canonical-corrected template sets of USPTO-50k and USPTO-460k. The canonical-corrected template sets lead to higher top-*N* accuracies, as well as faster training of the policy network, as shown in Table 2. We then used the retrained policy network to perform a Monte Carlo tree search-based retrosynthesis pathway search for the 100 ChEMBL compounds used as the benchmark in ref (27), for which AiZynthfinder trained on 1.2 M reactions from USPTO produced valid pathways for 55 compounds.^27^ For USPTO-50k, we found pathways for 34 and 41 molecules for the regular and canonical-corrected template sets, respectively. For USPTO-460k, we found pathways for 42 and 52 molecules for the regular and canonical-corrected template sets, respectively, as shown in Table 2. Correcting template sets for duplicates and nonexclusive templates therefore not only considerably increases the performance of single-step retrosynthesis models as highlighted in previous sections but also for multistep retrosynthesis approaches. With only 460,000 reactions, we obtain nearly the same amount of resolved pathways as the models trained on 1.2 M reactions in ref (27). We note that the canonical-corrected template sets describe the exact same chemical transformations as the regular template sets. The performance boost therefore comes solely from a better template ranking, and thus a better policy in the Monte Carlo tree search, highlighting the importance of the presented template correction algorithm. The processed template and model files are available online.^15^

**Table 2:** Performance of Policy Neural Network and Retrosynthesis Search of a Retrained AiZynthfinder Model Using Template Sets Reported in This Study^a^

	USPTO-50k reg	USPTO-50k can-cor	USPTO-460k reg	USPTO-460k can-cor
**Policy model**				
Top-10-accuracy (%)	83.9	88.5	87.5	89.2
Training time per epoch (s)	2	1	63	40

**Retrosynthesis search**				
Resolved pathways	34	41	42	52
Search time per pathway (s)	69	62	65	58

a Regular or canonical-corrected templates in the retrodirection from USPTO-50k or USPTO-460k.

## Limitations

We note that although the presented template correction and deduplication approach increases model performance for reaction prediction, as well as single-step and multistep retrosynthesis considerably, it suffers from the same problems as all template-based approaches.

Namely, templates small enough to efficiently train recommendation models may miss important functional groups further away from the reactive center, which are necessary for the reaction to proceed. Some of these relations might be learned by the template recommendation model but often only to a very general extent. We note that the template canonicalization and correction approach developed in this study does not introduce new, more general templates to a set but instead removes more specific or duplicate templates that are already covered by the set. This might remove information about functional groups further away from the reaction center for some templates but only if the reaction is also known to proceed without these functional groups.

A further general limitation of template-based models is their inability to learn or explore transformations unknown to the template set. Also, correct atom mappings of reactions are an important prerequisite for template extraction, which can be cumbersome to create.

Template-free retrosynthesis approaches^45−47^ alleviate some of these limitations but introduce others, such as limited interpretability as to why a specific transformation was suggested or which known reactions correspond to a suggested transformation. We therefore believe that even given the limitations of template-based approaches, it is still worthwhile to extend, optimize, and explore template-based retrosynthesis and forward prediction, alongside template-free approaches.

## Conclusion

We developed new canonicalization and hierarchical template correction algorithms as well as systematically studied the influence of template size, canonicalization, and exclusivity on the performance of various template-ranking algorithms. We find that duplicate and nonexclusive templates significantly impact the performance of all models across different template sizes for reaction prediction, single-step retrosynthesis, and multistep retrosynthesis. The number of nonexclusive templates is especially high in templates including special groups or large radii. Large performance boosts in both applicability and top-*N* accuracy for machine learning and heuristic models, as well as multistep retrosynthesis approaches, can be achieved by hierarchically correcting template sets for exclusivity. Smaller increases in performance can also be achieved by canonicalizing templates. The correction algorithm can furthermore be used to prune unnecessary special groups from templates automatically, which reduces the need of human interaction, and is thus an important step toward the automated curation of high-quality, exclusive, template sets.

## Data and Software Availability

The hierarchical correction code is available as a Python package on Github,^15^ along with the processed USPTO-50k and USPTO-460k data sets as CSV files and the complete set of Python scripts to reproduce all results in this study. The repository furthermore contains the AiZynthFinder template sets and policy models for USPTO-50k and USPTO-460k. The canonicalization code is available on Gitlab^16^ as part of the RDChiral C++ package. The modified RDChiral version to produce radius 0–3 templates without special groups is available on Github.^23^

## Supporting Information Available

Model hyperparameters, examples of extracted templates, top-*N* accuracies for reaction rules with explicit hydrogens, pseudocode representations of the hierarchical correction and canonicalization algorithms, supplemental figures (top-*N* accuracies and applicabilities for *N* = 1 and 50 for USPTO-50k, analogous figures for USPTO-460k), table of top-*N* accuracies and applicabilities of all systems (PDF)
