# Supplementary material for: Influence of Template Size, Canonicalization, and Exclusivity for Retrosynthesis and Reaction Prediction Applications
Source: J Chem Inf Model. 2021 Dec 23;62(1):16–26. doi: 10.1021/acs.jcim.1c01192 (PMC8757433; doi:10.1021/acs.jcim.1c01192)
Supplement: Supplementary file 1 — ci1c01192_si_001.pdf [file ci1c01192_si_001.pdf]

Supporting Information: Influence of Template Size, Canonicalization, and Exclusivity for Retrosynthesis and Reaction Prediction Applications

Esther Heid, Jiannan Liu, Andrea Aude, and William H. Green*

Department of Chemical Engineering, Massachusetts Institute of Technology, Cambridge, Massachusetts 02139, United States

E-mail: whgreen@mit.edu

S1 Model hyperparameters

Similarity model: Hyperparameters were taken from Ref. 1: Similarity between molecules was evaluated using Tanimoto similarities of Morgan count fingerprints at radius 2, with chirality and features, as implemented in RDKit.[2]

ML-fixed model: Hyperparameters were taken from Ref. 3. The input of the model corresponds to Morgan bit fingerprints of length 2048, at radius 2, with chirality, without features. We alternatively tested the performance of radius 3, especially for templates at radius 3, but did not notice significant differences in performance. The model architecture corresponds to a feed-forward neural net with one hidden layer of size 2048 and RELU activation function, trained with a batch size of 512, class weighting, a dropout rate of 0.2, a learning rate of 0.001, and early stopping.

ML-learned model: Hyperparameters were taken from Ref. 4, namely encoding of the molecular graph at depth 3 with mean aggregation, and a subsequent feed-forward neural network with two hidden layers. The hidden size in both the graph convolution and the feed-forward neural network was set to 2048. Smaller hidden sizes led to considerably poorer performance (evaluated for a hidden size of 300 on USPTO-50k, as well as a hidden size of 300 for the convolution and 2048 for the feed-forward neural network on USPTO-50k).Training was carried out with a batch size of 50, without dropout, and an increase in the learning rate from 10^{-4} to 10^{-3} within the first two epochs, then an exponential decrease to 10^{-4}. To investigate the reasons for the weaker performance of the ML-learned compared to the ML-fixed model we furthermore computed ML-learned models with a Morgan fingerprint appended to the output of the graph convolution. This boosts the performance considerably, but not beyond the performance of the ML-fixed model. Thus, we attribute the poor performance of the ML-learned model to two effects: First, the model is trained in an end-to-end fashion on two tasks, the embedding of the molecular graph to a molecular fingerprint, and the correlation of a fingerprint with a certain template. For large template sets, where many templates are not well populated, the first task becomes very difficult, and the extracted

fingerprints do not generalize well, *i.e.* perform worse than a Morgan fingerprint on a test set. If a Morgan fingerprint is concatenated to the learned fingerprint, the model performs better, but is still slightly worse compared to the ML-fixed model, which we attribute to the larger set of parameters, which again is prone to over-fitting.

S2 Examples of extracted templates

Fig. S1 depicts examples of reactions leading to the same or different templates based on the inclusion of special groups. If both templates (size 'Default') are kept in the template knowledge base, their non-exclusivity causes the performance of template ranking algorithms to deteriorate. A similar effect can be observed with templates at radius 2 and 3, as depicted in Fig. S2, which lists examples of templates (labelled 'actual template') that lead to the correct precursor but are not the same as the extracted, true template (labelled 'template goal'), and are thus not mutually exclusive. Non-exlusivity furthermore causes a discrepancy between evaluating ranks via identifying the correct template or the correct precursor, so that all examples in Fig. S2 would lead to a worse top-N-accuracy via template evaluation than via precursor evaluation.

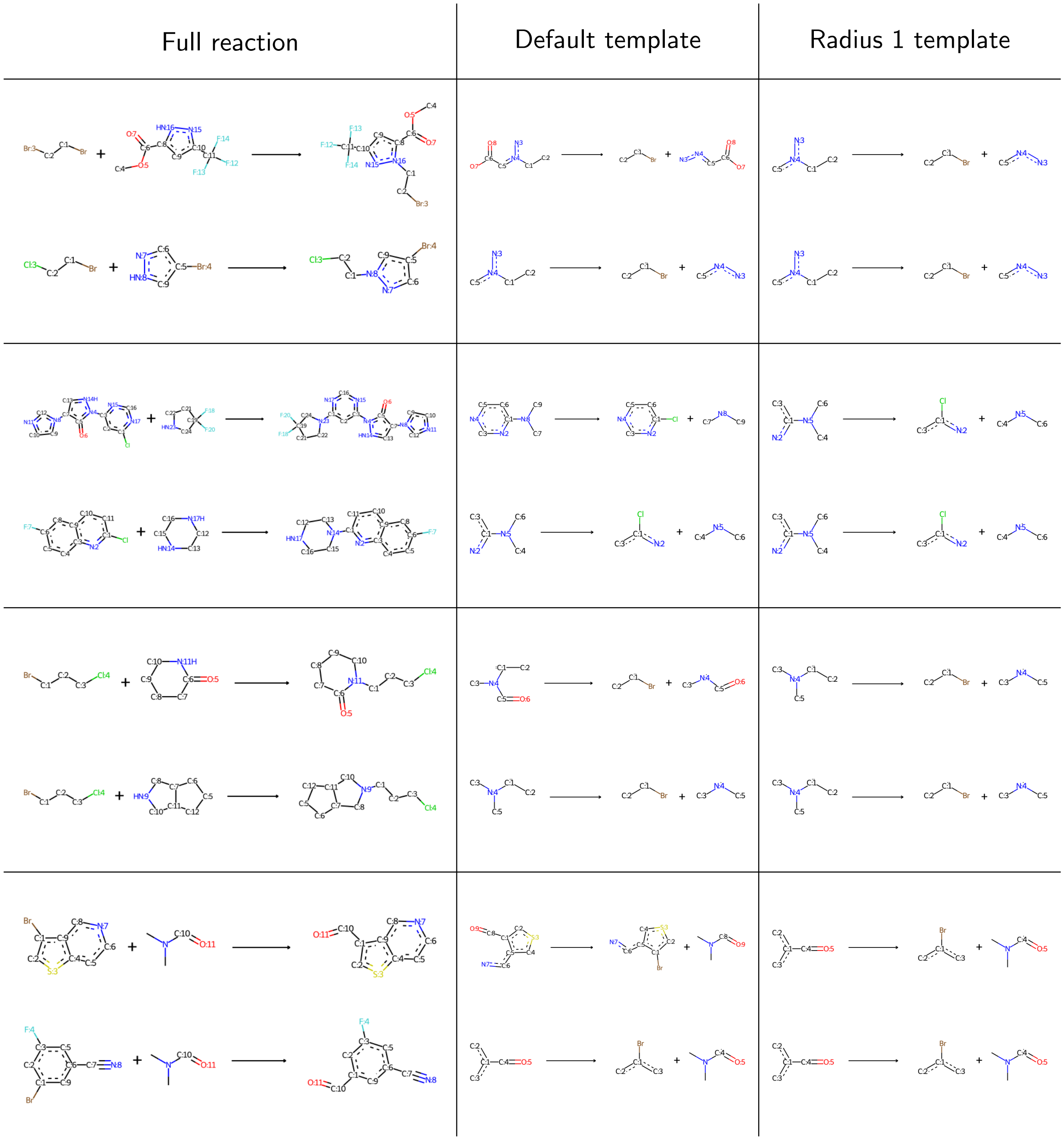

Figure S1: Examples of template extraction where radius 1 (no special groups) leads to similar templates, but the default parameters yield different reaction templates.

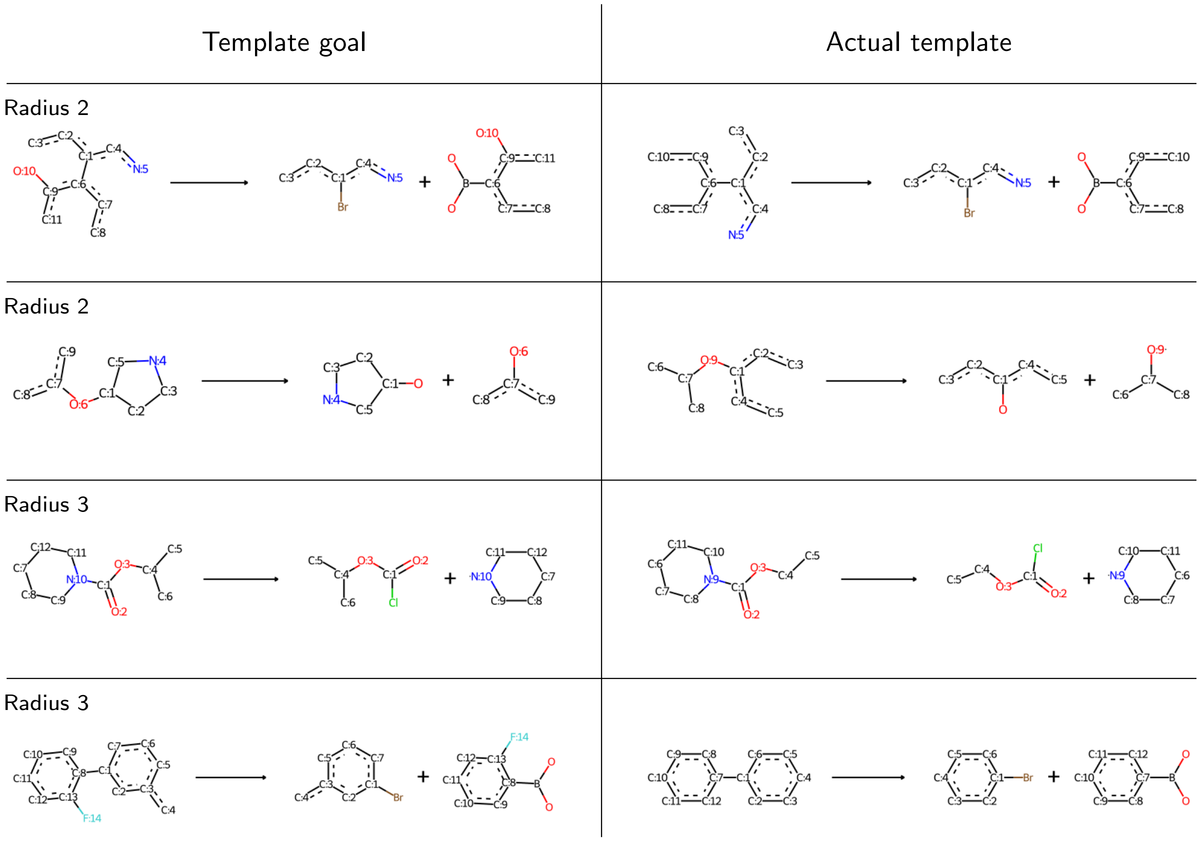

Figure S2: Demonstration of the branching issue with radius 2 and radius 3 templates.

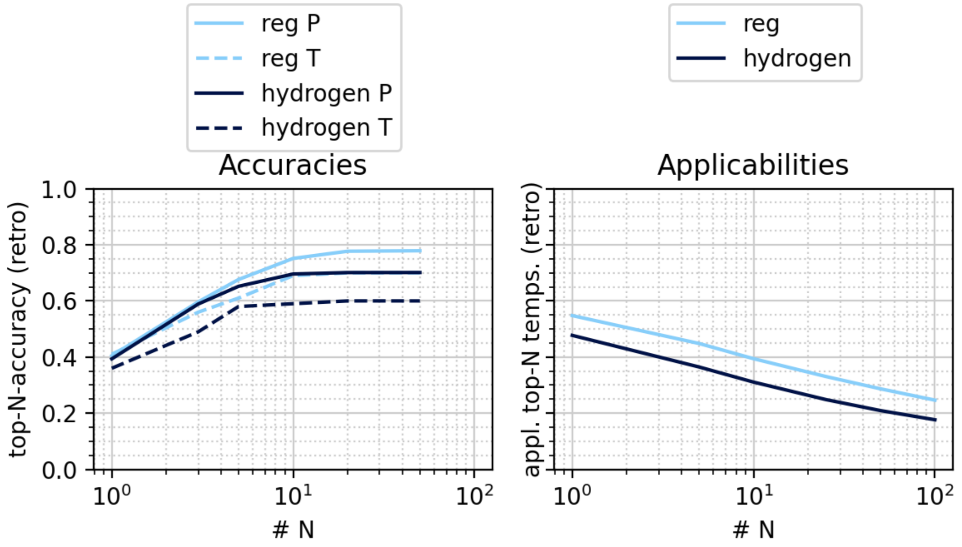

Figure S3: Top-N-accuracies (left) and applicabilities (right) of proposed retrosynthetic disconnections for the USPTO-50k dataset, similarity model. Comparison between default templates ('reg') and templates of the same size, with hydrogens specified for all atoms ('hydrogen').

S3 Inclusion of hydrogens to reaction rules

An alternative means to promote the exclusivity of templates is the inclusion of hydrogens at all template atoms. However, this added specificity decreases the top-N-accuracy and applicability, as shown in Fig. S3 exemplary for templates at radius 1 with special groups (default). It furthermore cannot resolve some exclusivity issues, such as those affiliated with special groups. We therefore did not pursue this approach further.

S4 Hierarchical template correction

A pseudocode representation of the template correction algorithm is given in Algorithm 1. For a list of templates, each template is compared to a list of most general templates to be kept (starting with an empty list). The template is kept if it is more general than another template. In that case, the more specific template is removed from the list. Parent-child relations are recorded to correctly replace the respective specific templates with more general

templates.

It is important to cluster the templates according to their reaction centers (minimal, most general templates) to avoid partial matches of the reaction center. Fig. S4 shows an example of two templates that produce a theoretically correct SMARTS pattern match, but encode different transformations with different leaving groups. Via the hierarchical, iterative correction of clustered templates, we avoid this unwanted behavior, since the two templates are never clustered into the same group due to the difference in reaction centers.

Algorithm 1 Correction of templates

```
function Sub(template1, template2)
    r1, p1 ← SplitToReacProd(template1)
    r2, p2 ← SplitToReacProd(template2)
    sub1to2 ← SubMatch(r2, r1) and SubMatch(p2, p1)
    sub2to1 ← SubMatch(r1, r2) and SubMatch(p1, p2)
    return (sub1to2, sub2to1)
end function

procedure CorrectTemplates(templates)
    unique ← MakeUniqueSortedList(templates)
    parent, child, duplicate ← empty dictionaries    ▷ dictionaries to record the current parent (more general), child (more specific) and duplicate templates
    include ←empty list
    for all templates tc in unique do
        used ← False
        for all templates ti in include do
            if Sub(tc, ti) equals (True, False) then
                remove ti from include
                add tc to include
                update child and parent
                used ← True
            else if Sub(tc, ti) equals (False, True) then
                update child and parent
                used ← True
                break loop
            else if Sub(tc, ti) equals (True, True) then
                update duplicate
                used ← True
                break loop
            end if
        end for
        if use is False then
            add tc to include
        end if
    end for
    corrected_templates ← empty list
    for all templates t in templates do
        replace t with duplicate or more general template if key t in duplicate or parent
        add new t to corrected_templates
    end for
    return corrected_templates
end procedure
```

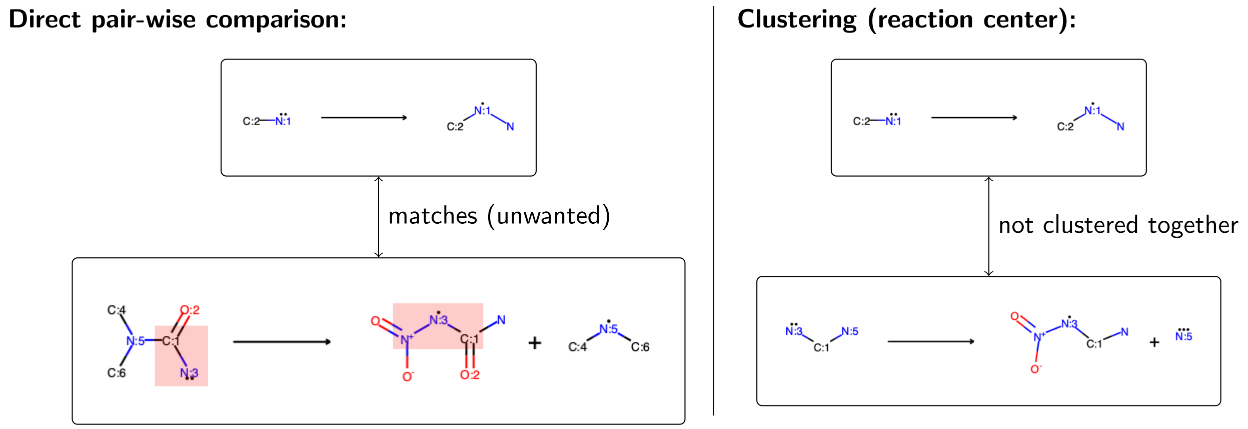

Figure S4: Left: Example of two templates that would produce an match of the SMARTS patterns which is technically correct but unwanted. Right: Minimal, most general templates for the two templates on the left show different reaction centers, so that the templates would be clustered into different groups and never compared directly.

S5 Canonicalization of templates

As described in the main article, the Weisfeiler-Lehman refinement was used as ranking method within our template canonicalization approach (LABEL function in Algorithm 2). Within the LABEL function, the COMBINE function is a simple label concatenation and compression in most cases, except for combining neighbor features, where a commutative and associative operation must be used. One can use numeric addition or sort the neighbor features before concatenation. The canonicalization process (Algorithm 2) starts with parsing the template and standardizing the atomic SMARTS in the template because they are used as features in the Weisfeiler-Lehman refinement method. In this study, we rely on RDKit to parse and write the SMARTS strings. Next, the reactant and the product graphs are merged according to their atom mapping numbers. Before the Weisfeiler-Lehman refinement, the CONNECTEDSUBGRAPHSIZE function computes the size of the subgraph, which serves as an invariant to distinguish highly symmetric loops. Then, the Weisfeiler-Lehman refinement is performed accompanied with chirality fixing. The chirality fixing removes unnecessary chiral tags due to the same neighbors around tetrahedral chiral centers or double bonds. After this point, the canonical form of the graph has already been generated, but additional steps are required for standardizing the atom mapping numbers if there is symmetry in the graph. The symmetry can be broken by assigning a unique and reproducible tag to the smallest non-unique node according to the order of the previously computed canonical labels. This tie-breaking process needs to be repeated until all the canonical labels are unique. At this time, the order of the canonical labels gives the canonical rank of the nodes and also their unique atom mapping numbers. In some applications, *e.g.* retrosynthesis planning, the mirror of a chiral template carries the same chemical transform due to the fact that R→S and S→R templates both indicate chirality inversion and the reaction templates may not selectively apply to only one of the enantiomers. In this scenario, one may simply choose the lexicographically smaller template string as the canonical form.

Algorithm 2 Canonicalization of templates

function LABEL(G, tag) ▷ Weisfeiler-Lehman refinement
  $label[v] \leftarrow$ **null**
  $counter \leftarrow 1$
  while canonical form of G based on $label[v]$ is not stable **And** $counter \leq |V|$ **do**
    $counter \leftarrow counter + 1$
    $label[v] \leftarrow$ COMBINE(FEATURE(v), $tag[v]$, $label[v]$, FEATURE(e_i), FEATURE(v_i), $label[v_i]$ for $(v_i, e_i) \in \mathcal{N}(v)$)
  end while
  return *label*
end function

procedure CANONIZETEMPLATE(*template*)
  $G_r, G_p \leftarrow$ PARSE(*template*) ▷ Graphs for the reactant and product.
  CANONIZEATOMICSMARTS(G_r)
  CANONIZEATOMICSMARTS(G_p)
  $G \leftarrow$ Merge G_r, G_p into a condensed graph using atom mapping.

  $tag[v] \leftarrow$ CONNECTEDSUBGRAPHSIZE(G) ▷ Compute the size of each connected subgraph using DFS
  repeat
    $label[v] \leftarrow$ LABEL(G, tag)
    Remove chiral tags for achiral atoms or double bonds according to *label*.
  until No chiral tags removed.

  $counter \leftarrow 1$
  while $label[v]$ not all unique **do** ▷ Tie-breaking
    SORT(*label*)
    $v' \leftarrow$ Find the first node with a non-unique label in $label[v]$.
    $tag[v'] \leftarrow$ COMBINE($tag[v'], counter$)
    $counter \leftarrow counter + 1$
    $label[v] \leftarrow$ LABEL(G, tag)
  end while

  $rank \leftarrow$ ARGSORT(*label*)
  ASSIGNCANONICALRANK($G, rank$)
  ASSIGNATOMMAPNUMBER($G, rank$)
  return SERIALIZE(G)
end procedure

S6 Supplemental figures

Top-N-accuracies: Fig. S5 and S6 depict top-N-accuracies for N = 1 and 50 for the USPTO-50k dataset. Top-N-accuracies for N = 1, 5 and 50 for the USPTO-460k dataset are shown in Fig. S7, S8 and S9. Fig. S10 depicts top-N-accuracies for the regular and canonical-corrected templates only for the ML-fixed model. Across the different datasets, N, and models, hierarchical correction of templates consistently leads to improved model performance and closes the gap between different evaluation schemes (identifying the correct template (darker shade) or precursor (lighter shade)). Canonicalizing templates also leads to a reduction in the gap between the two evaluation schemes, but to a smaller extent. Larger templates cause the ranking models to perform worse across all models (similarity, ML-fixed and ML-learned). Fig. S11 depicts top-N-accuracies for USPTO-460k via rank evaluation either per template (left), or per precursor (right), where one template might produce multiple precursors. The latter case is more relevant to multi-step synthesis pathway planning, since a too large number of precursors in each step makes an iterative search computationally expensive. Templates at radius 0 are readily applicable and produce a high amount of precursors, thus decreasing the top-N-accuracies per precursor even below the respective results at radius 1 or default templates. The same effect was observed for USPTO-50k in the main article.

Applicabilities of highest ranking templates: Applicabilities of the top 1 and 50 templates with the USPTO-50k dataset are shown in Fig. S12 and S13. For USPTO-460k, applicabilities of the top 1, 5 and 50 templates are depicted in Fig. S14, S15 and S16. For all datasets, models and number of evaluated templates, larger radii decrease applicability. Hierarchically corrected templates help the model to identify more applicable templates, especially at large template sizes ('Default', 'Radius 2' and 'Radius 3'). The influence of canonicalization is negligible.

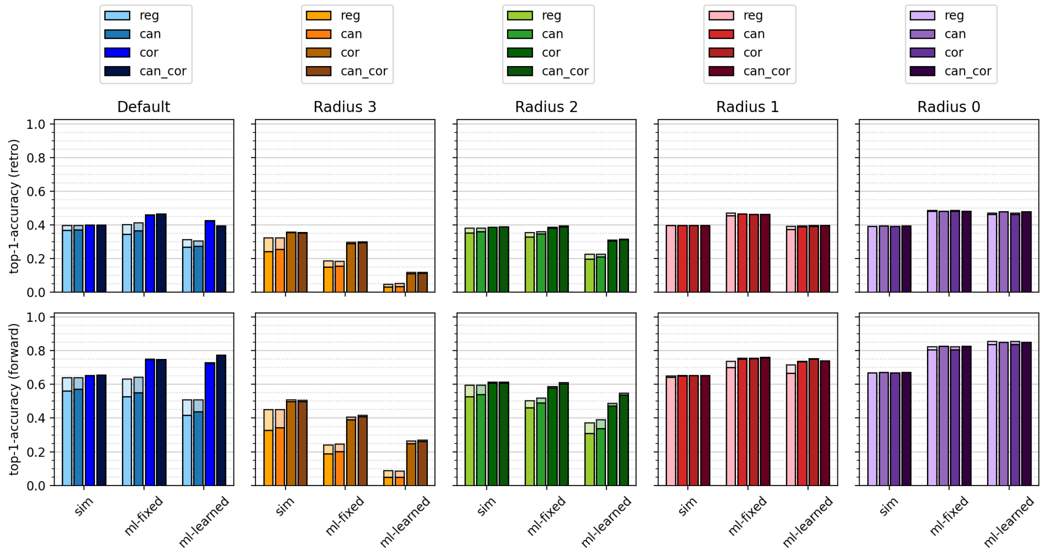

Figure S5: Top-1-accuracies of proposed retrosynthetic disconnections (top) and forward predictions (bottom) for the USPTO-50k dataset using the 'sim', 'ml-fixed', and 'ml-learned' models. The darker shade in a bar corresponds to evaluation via comparing templates, the lighter shade to comparing precursors or products. Each set of four bars shows the effects of canonicalizing ('can') or hierarchically corecting ('cor') the regular uncorrected templates ('reg'), or both ('can-cor').

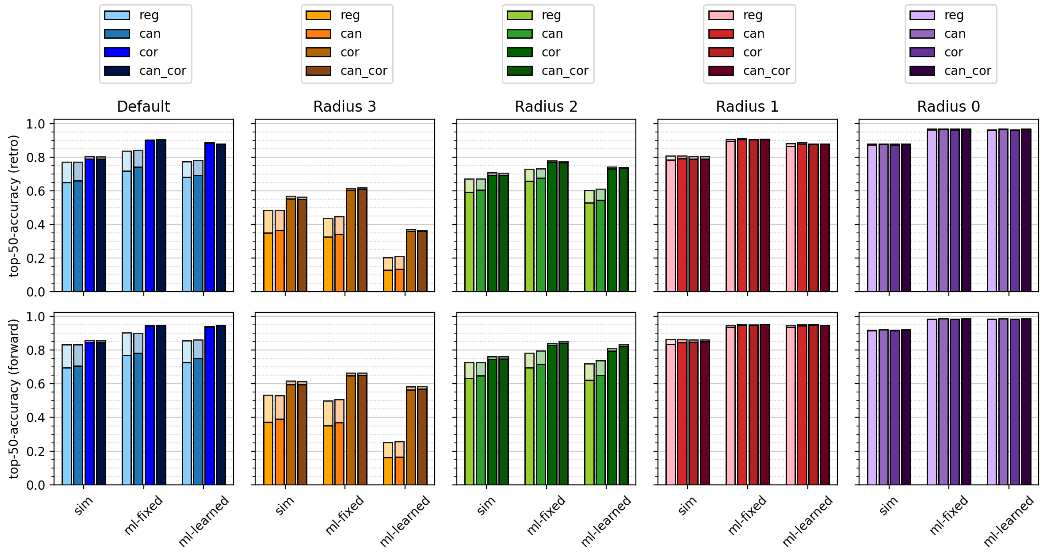

Figure S6: Top-50-accuracies of proposed retrosynthetic disconnections (top) and forward predictions (bottom) for the USPTO-50k dataset using the 'sim', 'ml-fixed', and 'ml-learned' models. The darker shade in a bar corresponds to evaluation via comparing templates, the lighter shade to comparing precursors or products. Each set of four bars shows the effects of canonicalizing ('can') or hierarchically corecting ('cor') the regular uncorrected templates ('reg'), or both ('can-cor')

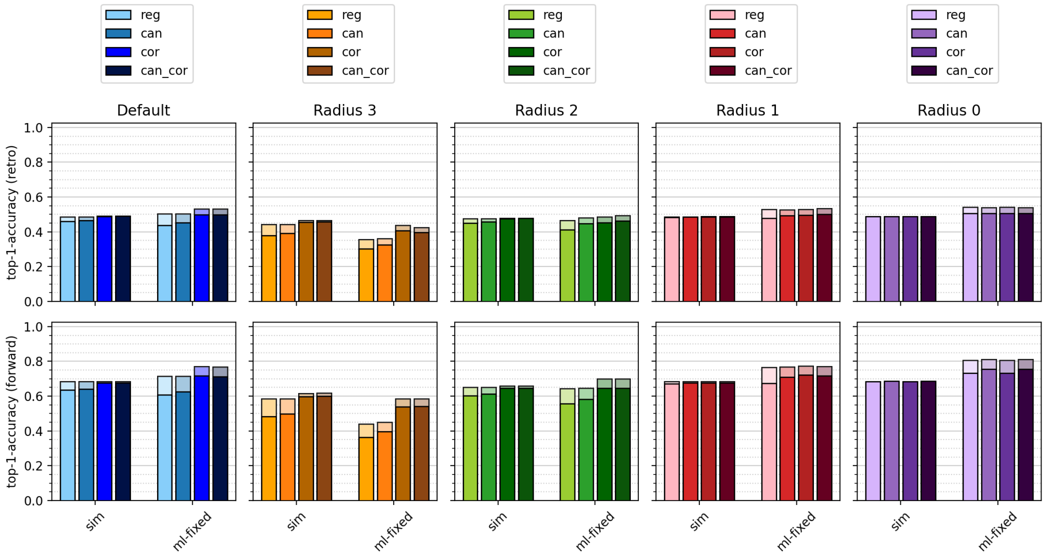

Figure S7: Top-1-accuracies of proposed retrosynthetic disconnections (top) and forward predictions (bottom) for the USPTO-460k dataset using the ‘sim’, ‘ml-fixed’, and ‘ml-learned’ models. The darker shade in a bar corresponds to evaluation via comparing templates, the lighter shade to comparing precursors or products. Each set of four bars shows the effects of canonicalizing (‘can’) or hierarchically corecting (‘cor’) the regular uncorrected templates (‘reg’), or both (‘can-cor’)

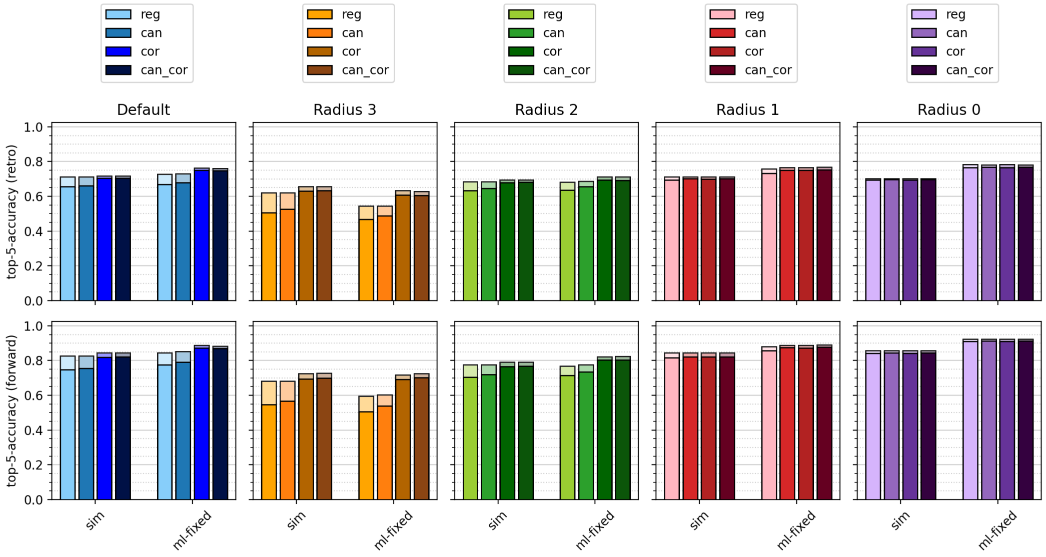

Figure S8: Top-5-accuracies of proposed retrosynthetic disconnections (top) and forward predictions (bottom) for the USPTO-460k dataset using the ‘sim’, ‘ml-fixed’, and ‘ml-learned’ models. The darker shade in a bar corresponds to evaluation via comparing templates, the lighter shade to comparing precursors or products. Each set of four bars shows the effects of canonicalizing (‘can’) or hierarchically corecting (‘cor’) the regular uncorrected templates (‘reg’), or both (‘can-cor’)

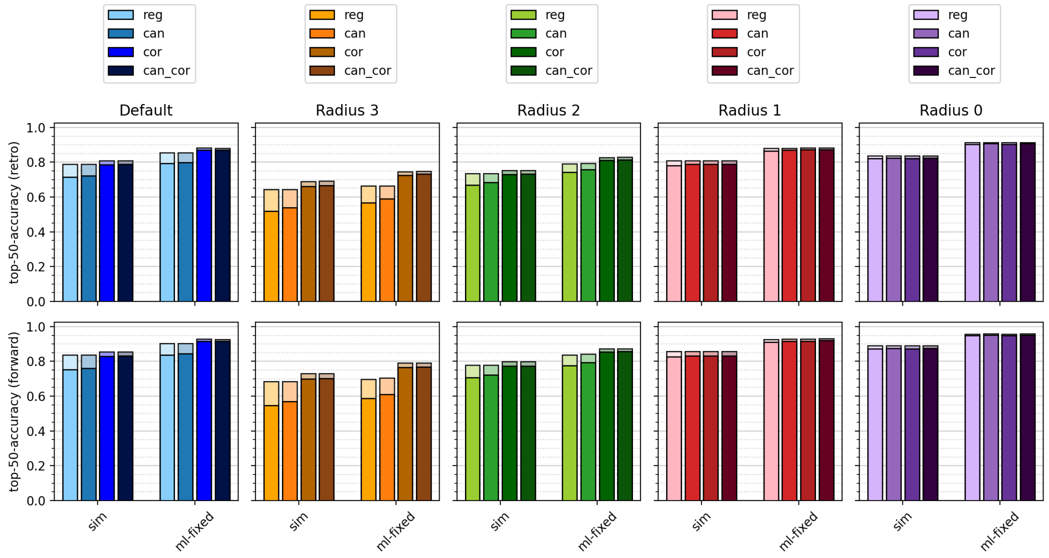

Figure S9: Top-50-accuracies of proposed retrosynthetic disconnections (top) and forward predictions (bottom) for the USPTO-460k dataset using the 'sim', 'ml-fixed', and 'ml-learned' models. The darker shade in a bar corresponds to evaluation via comparing templates, the lighter shade to comparing precursors or products. Each set of four bars shows the effects of canonicalizing ('can') or hierarchically corecting ('cor') the regular uncorrected templates ('reg'), or both ('can-cor')

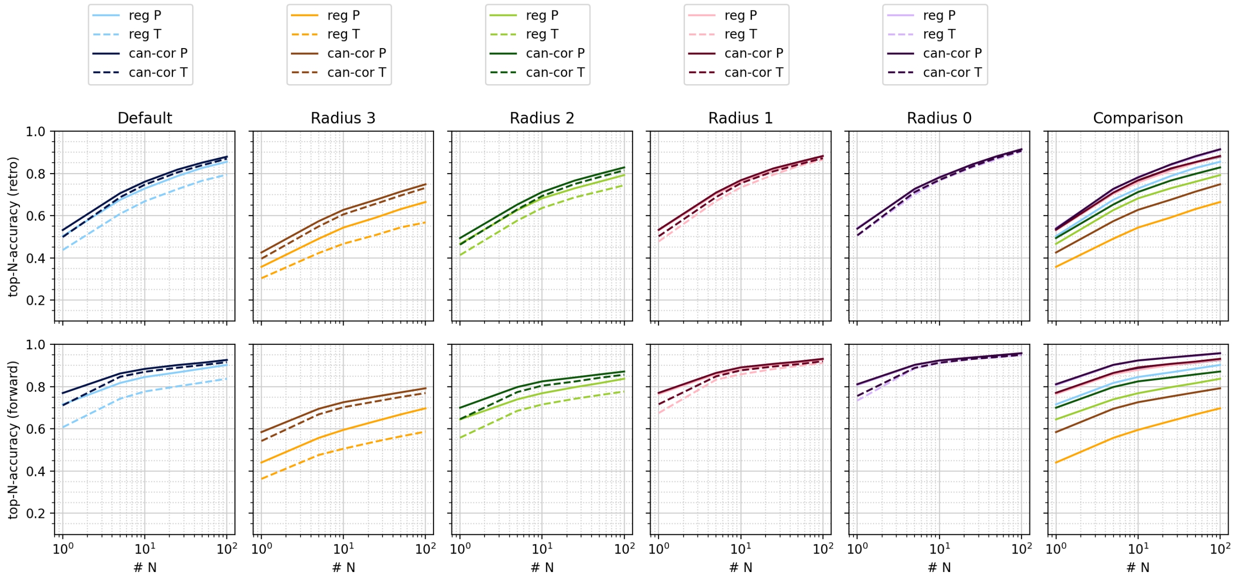

Figure S10: Dependence of top-N-accuracies of proposed retrosynthetic disconnections (top) and forward predictions (bottom) on the template scheme for the USPTO-460k dataset, ML-fixed model. 'reg' corresponds to uncorrected, 'can-cor' to canonical and corrected templates. P means evaluated by precursors or products (continuous line), T means evaluated by template match (dashed line).

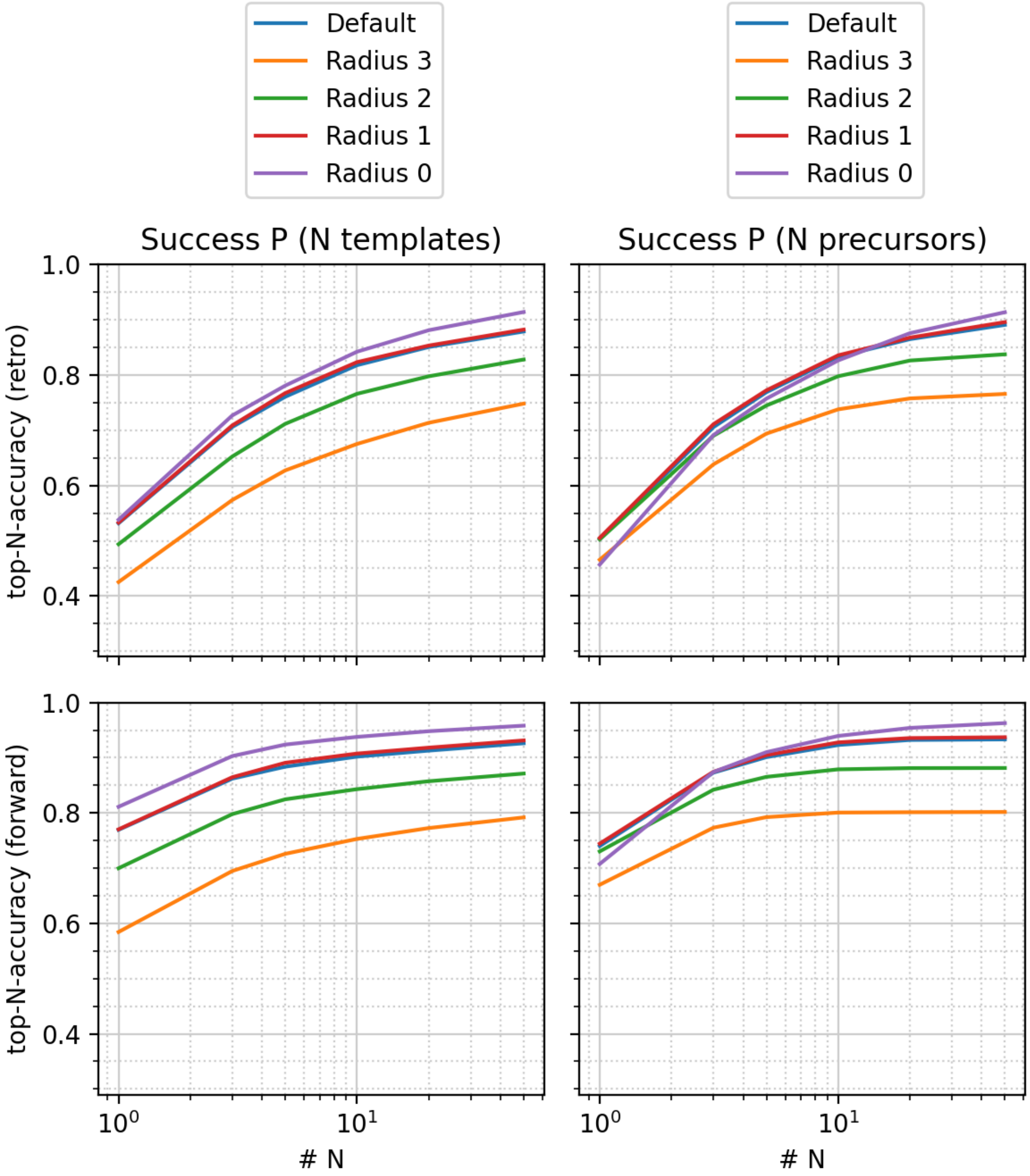

Figure S11: Top-N-accuracies of proposed retrosynthetic disconnections (top) and forward predictions (bottom) for the USPTO-460k dataset, canonical-corrected templates, ranking via the ML-fixed model. Left: Ranking via the number of templates, right: ranking via the number of precursors.

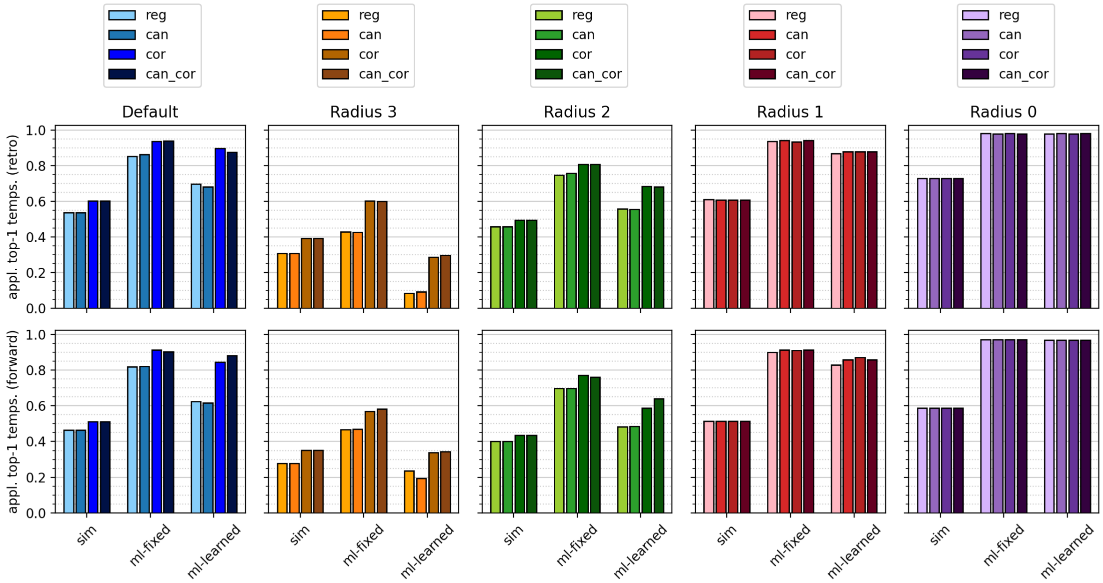

Figure S12: Fraction of applicable templates of the one highest ranked templates of proposed retrosynthetic disconnections (top) and forward predictions (bottom) for the USPTO-50k dataset using the 'sim', 'ml-fixed', and 'ml-learned' models. Each set of four bars shows the effects of canonicalizing ('can') or hierarchically corecting ('cor') the regular uncorrected templates ('reg'), or both ('can-cor').

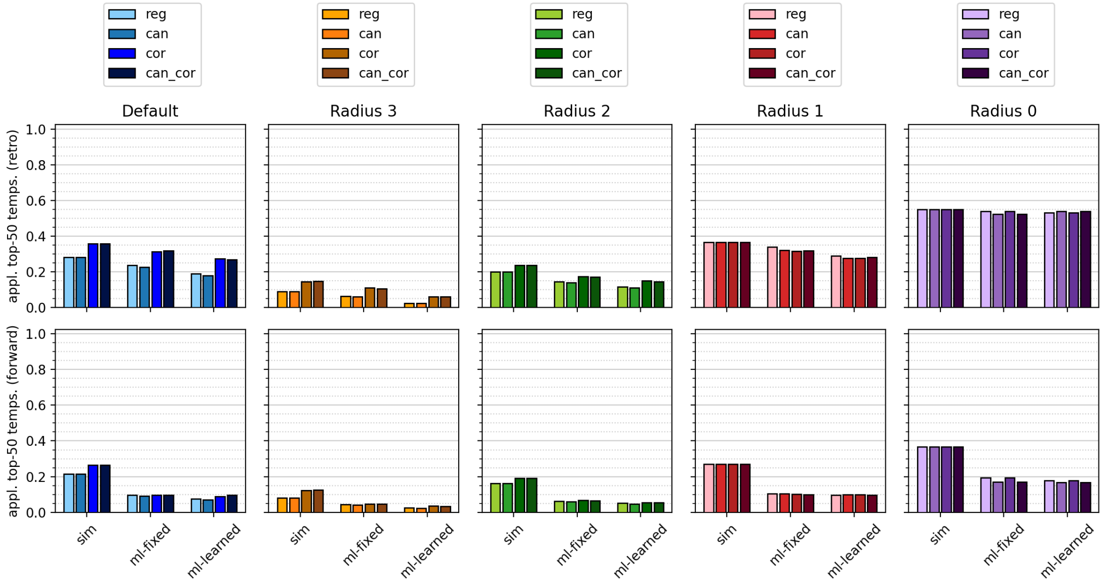

Figure S13: Fraction of applicable templates of the 50 highest ranked templates of proposed retrosynthetic disconnections (top) and forward predictions (bottom) for the USPTO-50k dataset using the 'sim', 'ml-fixed', and 'ml-learned' models. Each set of four bars shows the effects of canonicalizing ('can') or hierarchically corecting ('cor') the regular uncorrected templates ('reg'), or both ('can-cor').

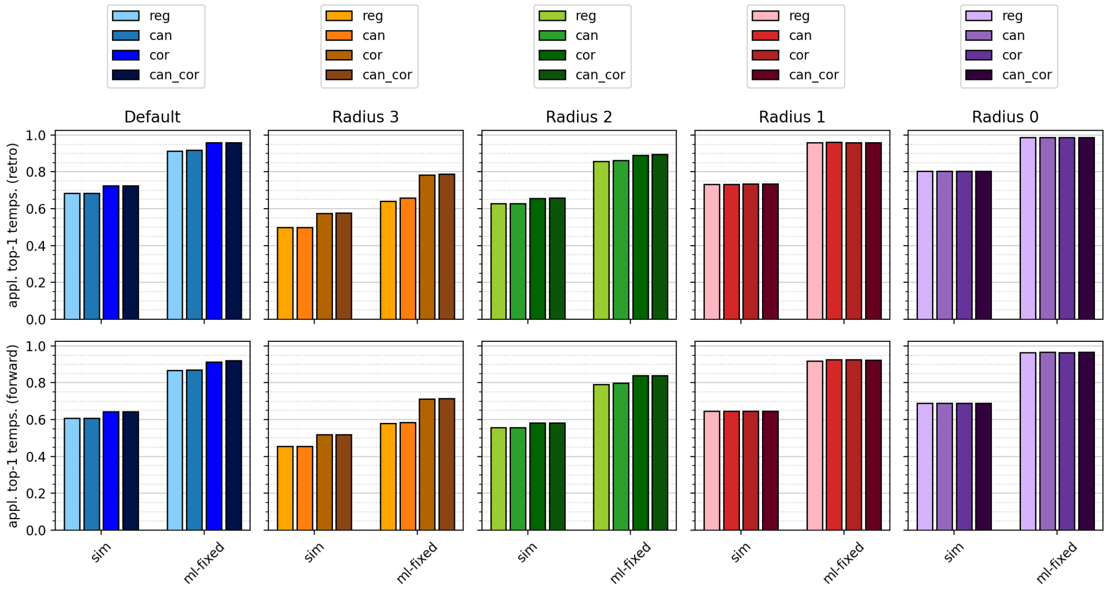

Figure S14: Fraction of applicable templates of the one highest ranked templates of proposed retrosynthetic disconnections (top) and forward predictions (bottom) for the USPTO-460k dataset using the 'sim', 'ml-fixed', and 'ml-learned' models. Each set of four bars shows the effects of canonicalizing ('can') or hierarchically corecting ('cor') the regular uncorrected templates ('reg'), or both ('can-cor').

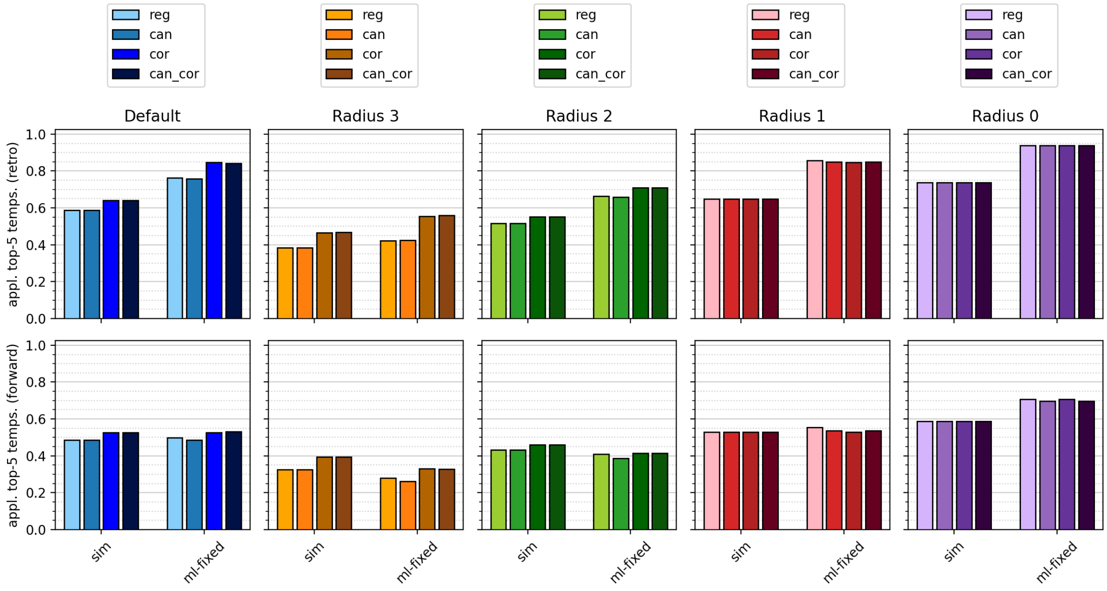

Figure S15: Fraction of applicable templates of the five highest ranked templates of proposed retrosynthetic disconnections (top) and forward predictions (bottom) for the USPTO-460k dataset using the 'sim', 'ml-fixed', and 'ml-learned' models. Each set of four bars shows the effects of canonicalizing ('can') or hierarchically corecting ('cor') the regular uncorrected templates ('reg'), or both ('can-cor').

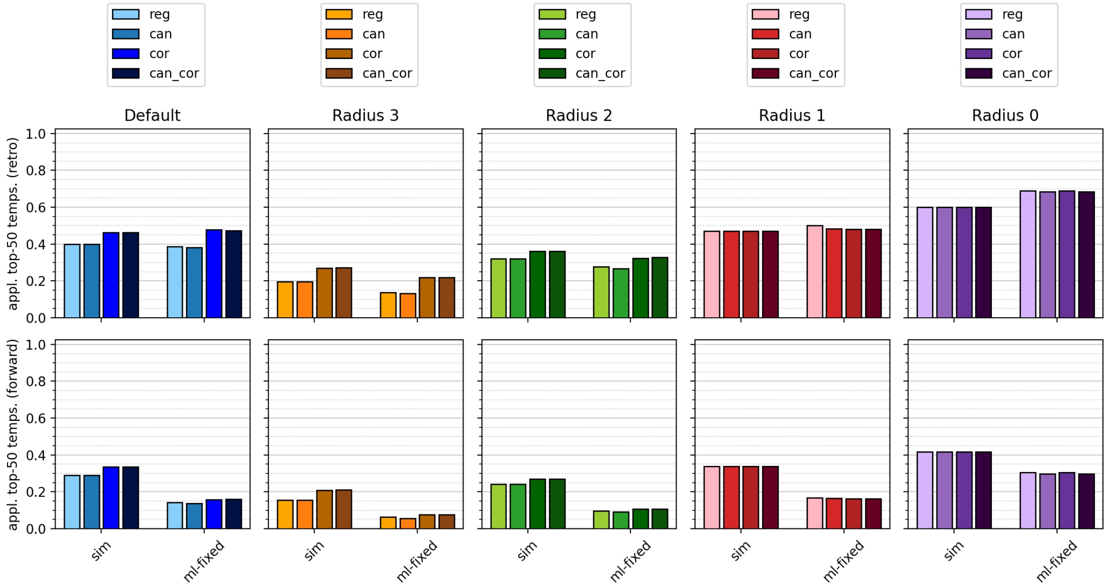

Figure S16: Fraction of applicable templates of the 50 highest ranked templates of proposed retrosynthetic disconnections (top) and forward predictions (bottom) for the USPTO-460k dataset using the 'sim', 'ml-fixed', and 'ml-learned' models. Each set of four bars shows the effects of canonicalizing ('can') or hierarchically corecting ('cor') the regular uncorrected templates ('reg'), or both ('can-cor').

S7 Top-N-accuracies of all systems

Table S1: Top-N-accuracies of all systems (in percent).

System	top-1	top-3	top-5	top-10	top-20	top-50
Similarity USPTO-50k/retro/default/T	36.8	52.3	58.3	63.4	64.8	64.9
Similarity USPTO-50k/retro/default/P	39.7	59.1	66.8	74.4	76.8	77.0
Similarity USPTO-50k/retro/default canonical/T	37.1	52.8	59.0	64.2	65.8	65.8
Similarity USPTO-50k/retro/default canonical/P	39.7	59.1	66.8	74.4	76.8	77.0
Similarity USPTO-50k/retro/default corrected/T	39.8	58.3	66.3	75.2	78.6	78.8
Similarity USPTO-50k/retro/default corrected/P	39.2	58.5	67.0	76.2	80.0	80.3
Similarity USPTO-50k/retro/default canonical corrected/T	39.9	58.4	66.3	75.2	78.6	78.8
Similarity USPTO-50k/retro/default canonical corrected/P	39.2	58.6	66.9	76.1	80.0	80.3
Similarity USPTO-50k/retro/radius 3/T	24.2	32.9	34.3	34.8	34.9	34.9
Similarity USPTO-50k/retro/radius 3/P	32.3	45.0	47.4	48.3	48.4	48.4
Similarity USPTO-50k/retro/radius 3 canonical/T	25.5	34.5	35.9	36.5	36.5	36.5
Similarity USPTO-50k/retro/radius 3 canonical/P	32.3	45.0	47.3	48.3	48.3	48.3
Similarity USPTO-50k/retro/radius 3 corrected/T	35.4	49.4	53.0	54.9	55.2	55.2
Similarity USPTO-50k/retro/radius 3 corrected/P	35.7	50.6	54.4	56.4	56.7	56.7
Similarity USPTO-50k/retro/radius 3 canonical corrected/T	35.3	48.9	52.9	54.6	54.9	54.9
Similarity USPTO-50k/retro/radius 3 canonical corrected/P	35.5	49.9	54.0	56.0	56.3	56.3
Similarity USPTO-50k/retro/radius 2/T	35.2	50.3	55.7	58.8	59.0	59.0
Similarity USPTO-50k/retro/radius 2/P	38.0	55.6	62.7	66.7	67.1	67.1
Similarity USPTO-50k/retro/radius 2 canonical/T	36.0	51.4	57.1	60.3	60.6	60.6
Similarity USPTO-50k/retro/radius 2 canonical/P	38.0	55.7	62.6	66.7	67.1	67.1
Similarity USPTO-50k/retro/radius 2 corrected/T	38.7	56.0	63.2	68.2	69.0	69.0
Similarity USPTO-50k/retro/radius 2 corrected/P	38.4	56.7	64.2	69.6	70.6	70.6
Similarity USPTO-50k/retro/radius 2 canonical corrected/T	38.8	56.0	63.3	68.3	69.1	69.1
Similarity USPTO-50k/retro/radius 2 canonical corrected/P	38.5	56.6	64.0	69.4	70.4	70.4
Similarity USPTO-50k/retro/radius 1/T	39.6	57.5	65.5	74.3	77.9	78.2
Similarity USPTO-50k/retro/radius 1/P	39.1	58.1	66.5	76.1	80.3	80.7
Similarity USPTO-50k/retro/radius 1 canonical/T	39.8	57.9	66.0	75.1	78.8	79.2
Similarity USPTO-50k/retro/radius 1 canonical/P	39.1	58.2	66.6	76.1	80.3	80.7
Similarity USPTO-50k/retro/radius 1 corrected/T	39.6	57.6	65.7	74.8	78.5	78.9
Similarity USPTO-50k/retro/radius 1 corrected/P	38.9	57.9	66.3	75.8	80.0	80.4
Similarity USPTO-50k/retro/radius 1 canonical corrected/T	39.6	57.7	65.7	74.8	78.5	78.8
Similarity USPTO-50k/retro/radius 1 canonical corrected/P	38.9	58.0	66.3	75.8	80.0	80.4
Similarity USPTO-50k/retro/radius 0/T	39.2	56.3	64.6	75.9	84.5	87.2
Similarity USPTO-50k/retro/radius 0/P	37.7	55.5	64.0	75.5	84.6	87.8
Similarity USPTO-50k/retro/radius 0 canonical/T	39.3	56.5	64.9	76.4	85.0	87.8

Continued on next page

Table S1 – continued from previous page

System	top-1	top-3	top-5	top-10	top-20	top-50
Similarity USPTO-50k/retro/radius 0 canonical/P	37.7	55.6	64.0	75.6	84.6	87.9
Similarity USPTO-50k/retro/radius 0 corrected/T	39.2	56.3	64.6	75.9	84.5	87.2
Similarity USPTO-50k/retro/radius 0 corrected/P	37.7	55.5	64.0	75.5	84.6	87.8
Similarity USPTO-50k/retro/radius 0 canonical corrected/T	39.3	56.5	64.9	76.4	85.0	87.8
Similarity USPTO-50k/retro/radius 0 canonical corrected/P	37.7	55.6	64.0	75.6	84.6	87.9
Similarity USPTO-50k/forward/default/T	56.3	68.2	69.4	69.6	69.6	69.6
Similarity USPTO-50k/forward/default/P	64.1	81.0	82.8	83.1	83.1	83.1
Similarity USPTO-50k/forward/default canonical/T	57.2	69.4	70.6	70.7	70.7	70.7
Similarity USPTO-50k/forward/default canonical/P	64.1	81.1	82.9	83.2	83.2	83.2
Similarity USPTO-50k/forward/default corrected/T	65.3	81.9	84.2	84.5	84.6	84.6
Similarity USPTO-50k/forward/default corrected/P	65.1	83.1	85.5	85.9	86.0	86.0
Similarity USPTO-50k/forward/default canonical corrected/T	65.6	82.2	84.4	84.7	84.8	84.8
Similarity USPTO-50k/forward/default canonical corrected/P	65.3	83.2	85.5	85.9	86.0	86.0
Similarity USPTO-50k/forward/radius 3/T	32.7	37.1	37.2	37.2	37.2	37.2
Similarity USPTO-50k/forward/radius 3/P	45.2	53.0	53.2	53.2	53.2	53.2
Similarity USPTO-50k/forward/radius 3 canonical/T	34.2	38.8	38.9	38.9	38.9	38.9
Similarity USPTO-50k/forward/radius 3 canonical/P	45.1	52.8	53.1	53.1	53.1	53.1
Similarity USPTO-50k/forward/radius 3 corrected/T	49.8	59.2	59.6	59.6	59.6	59.6
Similarity USPTO-50k/forward/radius 3 corrected/P	50.9	61.2	61.7	61.7	61.7	61.7
Similarity USPTO-50k/forward/radius 3 canonical corrected/T	49.9	59.2	59.7	59.7	59.7	59.7
Similarity USPTO-50k/forward/radius 3 canonical corrected/P	50.7	60.8	61.3	61.4	61.4	61.4
Similarity USPTO-50k/forward/radius 2/T	52.7	62.6	63.2	63.3	63.3	63.3
Similarity USPTO-50k/forward/radius 2/P	59.6	71.9	72.8	72.8	72.8	72.8
Similarity USPTO-50k/forward/radius 2 canonical/T	54.1	64.3	64.9	64.9	64.9	64.9
Similarity USPTO-50k/forward/radius 2 canonical/P	59.6	72.0	72.8	72.9	72.9	72.9
Similarity USPTO-50k/forward/radius 2 corrected/T	60.8	73.3	74.4	74.5	74.5	74.5
Similarity USPTO-50k/forward/radius 2 corrected/P	61.4	74.8	76.0	76.1	76.1	76.1
Similarity USPTO-50k/forward/radius 2 canonical corrected/T	61.0	73.6	74.7	74.8	74.8	74.8
Similarity USPTO-50k/forward/radius 2 canonical corrected/P	61.4	74.8	76.0	76.0	76.0	76.0
Similarity USPTO-50k/forward/radius 1/T	64.4	80.6	83.0	83.5	83.6	83.6
Similarity USPTO-50k/forward/radius 1/P	65.2	83.1	85.9	86.5	86.5	86.5
Similarity USPTO-50k/forward/radius 1 canonical/T	65.2	81.8	84.2	84.7	84.7	84.7
Similarity USPTO-50k/forward/radius 1 canonical/P	65.2	83.2	85.9	86.5	86.5	86.5
Similarity USPTO-50k/forward/radius 1 corrected/T	65.2	81.7	84.2	84.7	84.8	84.8
Similarity USPTO-50k/forward/radius 1 corrected/P	65.0	82.8	85.6	86.1	86.2	86.2
Similarity USPTO-50k/forward/radius 1 canonical corrected/T	65.5	82.1	84.4	84.9	85.0	85.0
Similarity USPTO-50k/forward/radius 1 canonical corrected/P	65.2	83.0	85.6	86.1	86.2	86.2
Similarity USPTO-50k/forward/radius 0/T	66.9	85.1	89.3	91.4	91.7	91.8

Continued on next page

Table S1 – continued from previous page

System	top-1	top-3	top-5	top-10	top-20	top-50
Similarity USPTO-50k/forward/radius 0/P	65.2	84.7	89.1	91.5	91.9	92.0
Similarity USPTO-50k/forward/radius 0 canonical/T	67.1	85.5	89.7	91.9	92.2	92.2
Similarity USPTO-50k/forward/radius 0 canonical/P	65.2	84.7	89.1	91.5	91.9	92.0
Similarity USPTO-50k/forward/radius 0 corrected/T	66.9	85.1	89.3	91.4	91.7	91.8
Similarity USPTO-50k/forward/radius 0 corrected/P	65.2	84.7	89.1	91.5	91.9	92.0
Similarity USPTO-50k/forward/radius 0 canonical corrected/T	67.1	85.5	89.7	91.9	92.2	92.2
Similarity USPTO-50k/forward/radius 0 canonical corrected/P	65.2	84.7	89.1	91.5	91.9	92.0
Similarity USPTO-460k/retro/default/T	46.2	61.0	65.5	69.8	71.2	71.5
Similarity USPTO-460k/retro/default/P	48.6	65.6	71.2	76.6	78.7	79.0
Similarity USPTO-460k/retro/default canonical/T	46.7	61.6	66.2	70.5	72.0	72.3
Similarity USPTO-460k/retro/default canonical/P	48.6	65.6	71.2	76.6	78.7	79.0
Similarity USPTO-460k/retro/default corrected/T	48.9	65.0	70.4	76.0	78.3	78.7
Similarity USPTO-460k/retro/default corrected/P	49.0	66.0	71.6	77.7	80.3	80.8
Similarity USPTO-460k/retro/default canonical corrected/T	49.0	65.1	70.5	76.1	78.4	78.8
Similarity USPTO-460k/retro/default canonical corrected/P	49.0	66.0	71.7	77.8	80.4	80.8
Similarity USPTO-460k/retro/radius 3/T	37.9	48.4	50.7	51.7	51.9	51.9
Similarity USPTO-460k/retro/radius 3/P	44.2	58.7	62.2	64.0	64.3	64.4
Similarity USPTO-460k/retro/radius 3 canonical/T	39.3	50.3	52.6	53.8	54.0	54.0
Similarity USPTO-460k/retro/radius 3 canonical/P	44.2	58.6	62.1	63.9	64.3	64.3
Similarity USPTO-460k/retro/radius 3 corrected/T	45.7	59.6	63.0	65.5	66.1	66.2
Similarity USPTO-460k/retro/radius 3 corrected/P	46.4	61.6	65.5	68.3	69.0	69.1
Similarity USPTO-460k/retro/radius 3 canonical corrected/T	45.8	59.8	63.3	65.8	66.4	66.6
Similarity USPTO-460k/retro/radius 3 canonical corrected/P	46.5	61.7	65.6	68.4	69.0	69.1
Similarity USPTO-460k/retro/radius 2/T	45.1	59.1	63.2	66.1	66.8	66.9
Similarity USPTO-460k/retro/radius 2/P	47.5	63.6	68.5	72.2	73.3	73.4
Similarity USPTO-460k/retro/radius 2 canonical/T	45.9	60.2	64.5	67.5	68.3	68.4
Similarity USPTO-460k/retro/radius 2 canonical/P	47.5	63.6	68.5	72.2	73.3	73.4
Similarity USPTO-460k/retro/radius 2 corrected/T	47.6	63.5	67.8	71.5	72.9	73.0
Similarity USPTO-460k/retro/radius 2 corrected/P	47.9	64.7	69.5	73.6	75.1	75.3
Similarity USPTO-460k/retro/radius 2 canonical corrected/T	47.7	63.6	68.1	71.7	73.1	73.2
Similarity USPTO-460k/retro/radius 2 canonical corrected/P	47.9	64.7	69.6	73.6	75.1	75.3
Similarity USPTO-460k/retro/radius 1/T	48.3	64.0	69.5	75.0	77.5	78.0
Similarity USPTO-460k/retro/radius 1/P	48.7	65.4	71.3	77.4	80.3	80.9
Similarity USPTO-460k/retro/radius 1 canonical/T	48.7	64.6	70.1	75.8	78.3	78.9
Similarity USPTO-460k/retro/radius 1 canonical/P	48.7	65.4	71.3	77.4	80.3	80.9
Similarity USPTO-460k/retro/radius 1 corrected/T	48.6	64.5	70.0	75.8	78.3	78.9
Similarity USPTO-460k/retro/radius 1 corrected/P	48.7	65.4	71.3	77.4	80.4	80.9
Similarity USPTO-460k/retro/radius 1 canonical corrected/T	48.7	64.6	70.1	75.8	78.4	78.9

Continued on next page

Table S1 – continued from previous page

System	top-1	top-3	top-5	top-10	top-20	top-50
Similarity USPTO-460k/retro/radius 1 canonical corrected/P	48.7	65.4	71.3	77.4	80.4	80.9
Similarity USPTO-460k/retro/radius 0/T	48.8	63.8	69.4	75.9	80.2	82.0
Similarity USPTO-460k/retro/radius 0/P	48.3	64.2	70.1	76.8	81.5	83.7
Similarity USPTO-460k/retro/radius 0 canonical/T	49.0	64.1	69.7	76.1	80.5	82.4
Similarity USPTO-460k/retro/radius 0 canonical/P	48.3	64.2	70.1	76.8	81.5	83.7
Similarity USPTO-460k/retro/radius 0 corrected/T	48.8	63.8	69.4	75.9	80.2	82.0
Similarity USPTO-460k/retro/radius 0 corrected/P	48.3	64.2	70.1	76.8	81.5	83.7
Similarity USPTO-460k/retro/radius 0 canonical corrected/T	49.0	64.1	69.7	76.1	80.5	82.4
Similarity USPTO-460k/retro/radius 0 canonical corrected/P	48.3	64.2	70.1	76.8	81.5	83.7
Similarity USPTO-460k/forward/default/T	63.7	73.5	74.9	75.4	75.4	75.4
Similarity USPTO-460k/forward/default/P	68.4	80.9	82.8	83.5	83.6	83.6
Similarity USPTO-460k/forward/default canonical/T	64.2	74.2	75.6	76.1	76.2	76.2
Similarity USPTO-460k/forward/default canonical/P	68.4	80.9	82.8	83.5	83.6	83.6
Similarity USPTO-460k/forward/default corrected/T	67.6	79.8	82.0	82.8	83.0	83.0
Similarity USPTO-460k/forward/default corrected/P	68.5	81.8	84.3	85.4	85.5	85.5
Similarity USPTO-460k/forward/default canonical corrected/T	67.7	79.9	82.1	82.9	83.1	83.1
Similarity USPTO-460k/forward/default canonical corrected/P	68.5	81.8	84.3	85.4	85.5	85.5
Similarity USPTO-460k/forward/radius 3/T	48.3	54.2	54.6	54.7	54.7	54.7
Similarity USPTO-460k/forward/radius 3/P	58.5	67.5	68.2	68.4	68.4	68.4
Similarity USPTO-460k/forward/radius 3 canonical/T	49.9	56.3	56.8	56.9	56.9	56.9
Similarity USPTO-460k/forward/radius 3 canonical/P	58.5	67.5	68.2	68.3	68.3	68.3
Similarity USPTO-460k/forward/radius 3 corrected/T	59.8	68.7	69.6	69.8	69.8	69.8
Similarity USPTO-460k/forward/radius 3 corrected/P	61.6	71.5	72.6	73.0	73.0	73.0
Similarity USPTO-460k/forward/radius 3 canonical corrected/T	60.1	69.0	69.8	70.1	70.1	70.1
Similarity USPTO-460k/forward/radius 3 canonical corrected/P	61.8	71.7	72.7	73.1	73.1	73.1
Similarity USPTO-460k/forward/radius 2/T	60.3	69.6	70.6	70.8	70.8	70.8
Similarity USPTO-460k/forward/radius 2/P	65.0	76.2	77.5	77.9	77.9	77.9
Similarity USPTO-460k/forward/radius 2 canonical/T	61.3	70.9	71.9	72.2	72.2	72.2
Similarity USPTO-460k/forward/radius 2 canonical/P	65.0	76.2	77.5	77.9	77.9	77.9
Similarity USPTO-460k/forward/radius 2 corrected/T	64.6	75.3	76.7	77.2	77.3	77.3
Similarity USPTO-460k/forward/radius 2 corrected/P	65.8	77.5	79.1	79.7	79.8	79.8
Similarity USPTO-460k/forward/radius 2 canonical corrected/T	64.7	75.3	76.8	77.3	77.4	77.4
Similarity USPTO-460k/forward/radius 2 canonical corrected/P	65.8	77.4	79.1	79.7	79.8	79.8
Similarity USPTO-460k/forward/radius 1/T	67.2	79.2	81.5	82.4	82.6	82.6
Similarity USPTO-460k/forward/radius 1/P	68.4	81.7	84.5	85.5	85.8	85.8
Similarity USPTO-460k/forward/radius 1 canonical/T	67.6	79.7	82.2	83.0	83.2	83.2
Similarity USPTO-460k/forward/radius 1 canonical/P	68.4	81.7	84.5	85.5	85.8	85.8
Similarity USPTO-460k/forward/radius 1 corrected/T	67.6	79.7	82.2	83.0	83.2	83.2

Continued on next page

Table S1 – continued from previous page

System	top-1	top-3	top-5	top-10	top-20	top-50
Similarity USPTO-460k/forward/radius 1 corrected/P	68.4	81.7	84.5	85.5	85.8	85.8
Similarity USPTO-460k/forward/radius 1 canonical corrected/T	67.7	79.8	82.2	83.1	83.3	83.3
Similarity USPTO-460k/forward/radius 1 canonical corrected/P	68.4	81.7	84.5	85.5	85.8	85.8
Similarity USPTO-460k/forward/radius 0/T	68.4	81.0	84.3	86.4	87.1	87.2
Similarity USPTO-460k/forward/radius 0/P	68.2	82.1	85.7	88.2	89.0	89.1
Similarity USPTO-460k/forward/radius 0 canonical/T	68.6	81.2	84.5	86.6	87.3	87.4
Similarity USPTO-460k/forward/radius 0 canonical/P	68.2	82.1	85.7	88.2	89.0	89.1
Similarity USPTO-460k/forward/radius 0 corrected/T	68.4	81.0	84.3	86.4	87.1	87.2
Similarity USPTO-460k/forward/radius 0 corrected/P	68.2	82.1	85.7	88.2	89.0	89.1
Similarity USPTO-460k/forward/radius 0 canonical corrected/T	68.6	81.2	84.5	86.6	87.3	87.4
Similarity USPTO-460k/forward/radius 0 canonical corrected/P	68.2	82.1	85.7	88.2	89.0	89.1
ML-fixed USPTO-50k/retro/default/T	34.4	51.4	57.8	63.7	67.8	71.7
ML-fixed USPTO-50k/retro/default/P	40.1	58.8	66.3	73.7	78.5	83.5
ML-fixed USPTO-50k/retro/default canonical/T	36.5	53.7	59.5	65.3	69.9	74.0
ML-fixed USPTO-50k/retro/default canonical/P	41.3	60.7	67.1	74.0	79.2	84.2
ML-fixed USPTO-50k/retro/default corrected/T	45.8	68.6	76.2	83.1	86.9	90.0
ML-fixed USPTO-50k/retro/default corrected/P	45.9	68.7	76.4	83.3	87.1	90.3
ML-fixed USPTO-50k/retro/default canonical corrected/T	46.4	68.2	76.0	82.9	86.8	90.3
ML-fixed USPTO-50k/retro/default canonical corrected/P	46.3	68.1	76.0	82.7	86.8	90.2
ML-fixed USPTO-50k/retro/radius 3/T	14.9	22.6	24.6	27.4	29.7	32.7
ML-fixed USPTO-50k/retro/radius 3/P	18.5	28.4	31.4	35.4	39.0	43.6
ML-fixed USPTO-50k/retro/radius 3 canonical/T	15.5	23.7	26.3	29.0	31.2	34.3
ML-fixed USPTO-50k/retro/radius 3 canonical/P	18.3	28.1	32.0	35.9	39.4	44.7
ML-fixed USPTO-50k/retro/radius 3 corrected/T	28.9	42.1	47.4	52.2	56.3	60.4
ML-fixed USPTO-50k/retro/radius 3 corrected/P	29.6	43.1	48.7	53.5	57.7	61.6
ML-fixed USPTO-50k/retro/radius 3 canonical corrected/T	29.5	43.3	48.2	53.4	57.0	60.9
ML-fixed USPTO-50k/retro/radius 3 canonical corrected/P	29.9	43.8	49.0	54.3	57.9	61.9
ML-fixed USPTO-50k/retro/radius 2/T	32.9	47.9	53.3	58.0	62.1	65.8
ML-fixed USPTO-50k/retro/radius 2/P	35.3	51.2	56.9	62.8	67.9	72.8
ML-fixed USPTO-50k/retro/radius 2 canonical/T	34.5	50.2	55.0	60.1	63.5	67.5
ML-fixed USPTO-50k/retro/radius 2 canonical/P	36.0	52.4	57.6	63.6	68.1	73.1
ML-fixed USPTO-50k/retro/radius 2 corrected/T	38.2	56.0	61.9	68.2	72.5	76.9
ML-fixed USPTO-50k/retro/radius 2 corrected/P	38.7	56.4	62.3	68.8	73.4	77.7
ML-fixed USPTO-50k/retro/radius 2 canonical corrected/T	38.9	55.8	61.9	67.9	72.3	76.8
ML-fixed USPTO-50k/retro/radius 2 canonical corrected/P	39.3	56.2	62.3	68.5	72.9	77.4
ML-fixed USPTO-50k/retro/radius 1/T	45.3	66.2	73.5	81.0	85.1	89.3
ML-fixed USPTO-50k/retro/radius 1/P	47.0	67.9	75.4	82.7	86.7	90.5
ML-fixed USPTO-50k/retro/radius 1 canonical/T	46.4	68.3	75.7	82.6	87.1	90.4

Continued on next page

Table S1 – continued from previous page

System	top-1	top-3	top-5	top-10	top-20	top-50
ML-fixed USPTO-50k/retro/radius 1 canonical/P	46.5	68.6	76.0	83.0	87.6	90.9
ML-fixed USPTO-50k/retro/radius 1 corrected/T	46.1	68.4	75.7	83.0	87.0	90.4
ML-fixed USPTO-50k/retro/radius 1 corrected/P	46.2	68.6	75.9	83.1	87.2	90.5
ML-fixed USPTO-50k/retro/radius 1 canonical corrected/T	46.2	68.0	75.6	83.2	87.5	90.7
ML-fixed USPTO-50k/retro/radius 1 canonical corrected/P	46.1	68.0	75.4	83.0	87.2	90.5
ML-fixed USPTO-50k/retro/radius 0/T	48.0	71.7	80.1	88.3	92.9	96.1
ML-fixed USPTO-50k/retro/radius 0/P	48.7	72.4	80.9	89.1	93.5	96.7
ML-fixed USPTO-50k/retro/radius 0 canonical/T	48.1	72.8	81.1	89.2	93.3	96.4
ML-fixed USPTO-50k/retro/radius 0 canonical/P	48.2	72.9	81.2	89.4	93.5	96.6
ML-fixed USPTO-50k/retro/radius 0 corrected/T	48.0	71.7	80.1	88.3	92.9	96.1
ML-fixed USPTO-50k/retro/radius 0 corrected/P	48.7	72.4	80.9	89.1	93.5	96.7
ML-fixed USPTO-50k/retro/radius 0 canonical corrected/T	48.1	72.8	81.1	89.2	93.3	96.4
ML-fixed USPTO-50k/retro/radius 0 canonical corrected/P	48.2	72.9	81.2	89.4	93.5	96.6
ML-fixed USPTO-50k/forward/default/T	52.7	65.5	68.2	71.7	74.2	77.1
ML-fixed USPTO-50k/forward/default/P	63.2	77.2	80.9	85.3	87.6	90.3
ML-fixed USPTO-50k/forward/default canonical/T	55.0	66.9	70.7	73.3	75.9	78.3
ML-fixed USPTO-50k/forward/default canonical/P	64.2	78.2	81.8	85.2	88.0	90.0
ML-fixed USPTO-50k/forward/default corrected/T	74.9	87.5	89.6	91.6	93.1	94.3
ML-fixed USPTO-50k/forward/default corrected/P	75.1	87.8	89.8	91.9	93.5	94.6
ML-fixed USPTO-50k/forward/default canonical corrected/T	75.0	87.6	90.0	91.8	93.4	94.9
ML-fixed USPTO-50k/forward/default canonical corrected/P	75.0	87.7	90.1	91.9	93.4	94.8
ML-fixed USPTO-50k/forward/radius 3/T	19.0	25.2	27.7	29.8	32.3	35.2
ML-fixed USPTO-50k/forward/radius 3/P	24.2	33.4	37.1	41.0	45.0	49.9
ML-fixed USPTO-50k/forward/radius 3 canonical/T	20.2	26.6	28.8	31.8	34.0	37.1
ML-fixed USPTO-50k/forward/radius 3 canonical/P	24.7	33.8	37.2	41.7	45.7	50.8
ML-fixed USPTO-50k/forward/radius 3 corrected/T	39.1	51.2	54.9	58.7	61.9	64.8
ML-fixed USPTO-50k/forward/radius 3 corrected/P	40.8	52.6	56.5	60.3	63.6	66.4
ML-fixed USPTO-50k/forward/radius 3 canonical corrected/T	40.9	52.3	55.8	59.4	62.0	65.0
ML-fixed USPTO-50k/forward/radius 3 canonical corrected/P	41.6	53.6	57.3	61.0	63.8	66.5
ML-fixed USPTO-50k/forward/radius 2/T	46.1	57.7	61.2	64.4	66.7	69.5
ML-fixed USPTO-50k/forward/radius 2/P	50.3	63.3	67.5	71.5	75.0	78.3
ML-fixed USPTO-50k/forward/radius 2 canonical/T	49.0	59.7	62.6	66.3	68.6	71.6
ML-fixed USPTO-50k/forward/radius 2 canonical/P	52.0	64.3	68.5	73.0	75.9	79.4
ML-fixed USPTO-50k/forward/radius 2 corrected/T	57.9	70.8	74.3	78.0	80.6	83.0
ML-fixed USPTO-50k/forward/radius 2 corrected/P	58.9	71.9	75.4	78.8	81.6	84.1
ML-fixed USPTO-50k/forward/radius 2 canonical corrected/T	60.4	72.9	76.0	79.6	82.0	84.3
ML-fixed USPTO-50k/forward/radius 2 canonical corrected/P	61.1	73.7	76.8	80.5	82.9	85.3
ML-fixed USPTO-50k/forward/radius 1/T	70.1	85.1	88.0	90.6	92.1	93.7

Continued on next page

Table S1 – continued from previous page

System	top-1	top-3	top-5	top-10	top-20	top-50
ML-fixed USPTO-50k/forward/radius 1/P	73.8	87.2	89.6	91.9	93.3	94.8
ML-fixed USPTO-50k/forward/radius 1 canonical/T	75.1	87.3	89.6	91.8	93.4	94.7
ML-fixed USPTO-50k/forward/radius 1 canonical/P	75.6	87.9	90.1	92.5	94.0	95.3
ML-fixed USPTO-50k/forward/radius 1 corrected/T	75.3	87.3	89.6	91.8	93.4	94.8
ML-fixed USPTO-50k/forward/radius 1 corrected/P	75.5	87.5	89.8	92.0	93.8	95.0
ML-fixed USPTO-50k/forward/radius 1 canonical corrected/T	75.9	87.6	90.1	92.3	93.8	95.2
ML-fixed USPTO-50k/forward/radius 1 canonical corrected/P	76.1	87.7	90.2	92.1	93.8	95.2
ML-fixed USPTO-50k/forward/radius 0/T	80.6	93.8	95.9	97.2	97.9	98.5
ML-fixed USPTO-50k/forward/radius 0/P	82.6	93.7	95.6	96.8	97.5	98.1
ML-fixed USPTO-50k/forward/radius 0 canonical/T	82.8	94.5	96.4	97.6	98.4	98.9
ML-fixed USPTO-50k/forward/radius 0 canonical/P	82.6	93.9	95.6	96.9	97.6	98.0
ML-fixed USPTO-50k/forward/radius 0 corrected/T	80.6	93.8	95.9	97.2	97.9	98.5
ML-fixed USPTO-50k/forward/radius 0 corrected/P	82.6	93.7	95.6	96.8	97.5	98.1
ML-fixed USPTO-50k/forward/radius 0 canonical corrected/T	82.8	94.5	96.4	97.6	98.4	98.9
ML-fixed USPTO-50k/forward/radius 0 canonical corrected/P	82.6	93.9	95.6	96.9	97.6	98.0
ML-fixed USPTO-460k/retro/default/T	43.7	60.9	66.8	72.5	76.6	79.4
ML-fixed USPTO-460k/retro/default/P	50.3	67.5	72.8	78.7	82.6	85.5
ML-fixed USPTO-460k/retro/default canonical/T	45.2	62.4	67.8	73.2	76.4	79.9
ML-fixed USPTO-460k/retro/default canonical/P	50.3	67.5	73.0	78.6	81.9	85.5
ML-fixed USPTO-460k/retro/default corrected/T	49.8	68.7	75.1	80.4	84.2	87.1
ML-fixed USPTO-460k/retro/default corrected/P	53.3	70.4	76.4	81.6	85.2	88.2
ML-fixed USPTO-460k/retro/default canonical corrected/T	49.9	68.7	74.7	80.5	83.9	87.0
ML-fixed USPTO-460k/retro/default canonical corrected/P	53.2	70.6	76.1	81.8	85.1	87.9
ML-fixed USPTO-460k/retro/radius 3/T	30.3	42.3	46.7	50.9	54.4	56.8
ML-fixed USPTO-460k/retro/radius 3/P	35.7	49.1	54.3	59.1	63.1	66.4
ML-fixed USPTO-460k/retro/radius 3 canonical/T	32.5	45.0	48.9	53.2	56.2	58.9
ML-fixed USPTO-460k/retro/radius 3 canonical/P	36.0	49.7	54.5	59.5	63.0	66.4
ML-fixed USPTO-460k/retro/radius 3 corrected/T	40.6	55.7	60.8	66.3	69.6	72.5
ML-fixed USPTO-460k/retro/radius 3 corrected/P	43.8	58.5	63.2	68.4	71.7	74.6
ML-fixed USPTO-460k/retro/radius 3 canonical corrected/T	39.6	54.9	60.6	65.7	69.6	73.1
ML-fixed USPTO-460k/retro/radius 3 canonical corrected/P	42.5	57.4	62.7	67.5	71.4	74.8
ML-fixed USPTO-460k/retro/radius 2/T	41.3	57.7	63.6	68.7	71.5	74.4
ML-fixed USPTO-460k/retro/radius 2/P	46.6	62.6	68.2	73.0	76.1	79.2
ML-fixed USPTO-460k/retro/radius 2 canonical/T	44.7	60.7	65.5	70.0	73.1	75.8
ML-fixed USPTO-460k/retro/radius 2 canonical/P	48.1	64.0	68.8	73.2	76.2	79.3
ML-fixed USPTO-460k/retro/radius 2 corrected/T	45.2	63.6	69.5	74.7	78.5	81.2
ML-fixed USPTO-460k/retro/radius 2 corrected/P	48.6	66.0	71.3	76.3	79.9	82.7
ML-fixed USPTO-460k/retro/radius 2 canonical corrected/T	46.3	63.0	69.3	75.0	78.3	81.5

Continued on next page

Table S1 – continued from previous page

System	top-1	top-3	top-5	top-10	top-20	top-50
ML-fixed USPTO-460k/retro/radius 2 canonical corrected/P	49.4	65.3	71.2	76.6	79.8	82.8
ML-fixed USPTO-460k/retro/radius 1/T	47.8	66.9	73.3	79.4	83.4	86.5
ML-fixed USPTO-460k/retro/radius 1/P	53.0	70.3	75.7	81.3	84.9	88.1
ML-fixed USPTO-460k/retro/radius 1 canonical/T	49.4	69.0	75.1	80.3	83.8	86.9
ML-fixed USPTO-460k/retro/radius 1 canonical/P	52.7	70.9	76.6	81.5	84.8	88.1
ML-fixed USPTO-460k/retro/radius 1 corrected/T	49.6	68.7	75.1	80.5	84.3	87.2
ML-fixed USPTO-460k/retro/radius 1 corrected/P	53.0	70.9	76.6	81.8	85.5	88.2
ML-fixed USPTO-460k/retro/radius 1 canonical corrected/T	50.2	68.8	75.3	81.0	84.2	87.2
ML-fixed USPTO-460k/retro/radius 1 canonical corrected/P	53.3	70.9	76.7	82.4	85.4	88.2
ML-fixed USPTO-460k/retro/radius 0/T	50.5	70.1	76.6	82.8	86.7	90.4
ML-fixed USPTO-460k/retro/radius 0/P	54.2	72.3	78.4	83.9	87.7	91.2
ML-fixed USPTO-460k/retro/radius 0 canonical/T	50.7	71.2	76.9	83.2	87.4	90.7
ML-fixed USPTO-460k/retro/radius 0 canonical/P	53.8	72.7	78.1	84.2	88.1	91.4
ML-fixed USPTO-460k/retro/radius 0 corrected/T	50.5	70.1	76.6	82.8	86.7	90.4
ML-fixed USPTO-460k/retro/radius 0 corrected/P	54.2	72.3	78.4	83.9	87.7	91.2
ML-fixed USPTO-460k/retro/radius 0 canonical corrected/T	50.7	71.2	76.9	83.2	87.4	90.7
ML-fixed USPTO-460k/retro/radius 0 canonical corrected/P	53.8	72.7	78.1	84.2	88.1	91.4
ML-fixed USPTO-460k/forward/default/T	60.7	74.3	77.7	80.1	81.9	83.7
ML-fixed USPTO-460k/forward/default/P	71.6	81.7	84.5	86.7	88.5	90.2
ML-fixed USPTO-460k/forward/default canonical/T	62.7	75.8	79.0	81.3	82.6	84.5
ML-fixed USPTO-460k/forward/default canonical/P	71.4	82.0	85.2	87.5	88.7	90.4
ML-fixed USPTO-460k/forward/default corrected/T	71.6	84.9	87.2	88.9	90.4	91.6
ML-fixed USPTO-460k/forward/default corrected/P	77.0	86.4	88.7	90.3	91.8	92.8
ML-fixed USPTO-460k/forward/default canonical corrected/T	71.1	84.6	87.0	88.9	90.2	91.5
ML-fixed USPTO-460k/forward/default canonical corrected/P	76.9	86.2	88.4	90.2	91.3	92.6
ML-fixed USPTO-460k/forward/radius 3/T	36.3	47.6	50.6	54.0	56.4	58.7
ML-fixed USPTO-460k/forward/radius 3/P	44.0	55.7	59.5	63.7	66.8	69.7
ML-fixed USPTO-460k/forward/radius 3 canonical/T	39.6	50.5	54.0	57.1	59.1	61.1
ML-fixed USPTO-460k/forward/radius 3 canonical/P	45.1	56.5	60.4	64.2	67.1	70.4
ML-fixed USPTO-460k/forward/radius 3 corrected/T	53.9	66.3	69.2	72.6	74.5	76.5
ML-fixed USPTO-460k/forward/radius 3 corrected/P	58.6	69.0	71.8	75.0	77.0	79.0
ML-fixed USPTO-460k/forward/radius 3 canonical corrected/T	54.2	66.9	70.2	73.0	75.1	76.9
ML-fixed USPTO-460k/forward/radius 3 canonical corrected/P	58.4	69.5	72.6	75.3	77.3	79.2
ML-fixed USPTO-460k/forward/radius 2/T	55.8	68.5	71.5	74.2	76.0	77.6
ML-fixed USPTO-460k/forward/radius 2/P	64.5	74.0	76.9	79.7	81.6	83.7
ML-fixed USPTO-460k/forward/radius 2 canonical/T	58.4	70.5	73.6	76.2	77.8	79.5
ML-fixed USPTO-460k/forward/radius 2 canonical/P	64.6	74.8	77.7	80.2	82.1	84.2
ML-fixed USPTO-460k/forward/radius 2 corrected/T	64.6	77.9	80.3	82.9	84.3	85.5

Continued on next page

Table S1 – continued from previous page

System	top-1	top-3	top-5	top-10	top-20	top-50
ML-fixed USPTO-460k/forward/radius 2 corrected/P	69.8	80.0	82.1	84.7	86.0	87.2
ML-fixed USPTO-460k/forward/radius 2 canonical corrected/T	64.6	77.4	80.4	82.4	84.2	85.6
ML-fixed USPTO-460k/forward/radius 2 canonical corrected/P	70.0	79.8	82.5	84.3	85.7	87.1
ML-fixed USPTO-460k/forward/radius 1/T	67.5	83.3	85.8	88.3	89.8	91.2
ML-fixed USPTO-460k/forward/radius 1/P	76.4	85.8	87.9	90.1	91.3	92.7
ML-fixed USPTO-460k/forward/radius 1 canonical/T	71.0	84.9	87.4	89.4	90.5	91.7
ML-fixed USPTO-460k/forward/radius 1 canonical/P	76.7	86.6	88.8	90.4	91.5	92.8
ML-fixed USPTO-460k/forward/radius 1 corrected/T	72.3	84.6	87.4	89.2	90.3	91.5
ML-fixed USPTO-460k/forward/radius 1 corrected/P	77.3	86.4	88.8	90.4	91.5	92.7
ML-fixed USPTO-460k/forward/radius 1 canonical corrected/T	71.6	84.8	87.7	89.5	90.6	92.0
ML-fixed USPTO-460k/forward/radius 1 canonical corrected/P	77.0	86.5	89.1	90.7	91.8	93.1
ML-fixed USPTO-460k/forward/radius 0/T	73.4	88.6	91.2	93.0	93.8	94.9
ML-fixed USPTO-460k/forward/radius 0/P	80.7	90.1	92.4	93.7	94.5	95.7
ML-fixed USPTO-460k/forward/radius 0 canonical/T	75.6	88.8	91.3	93.1	94.0	95.0
ML-fixed USPTO-460k/forward/radius 0 canonical/P	81.1	90.3	92.4	93.8	94.8	95.8
ML-fixed USPTO-460k/forward/radius 0 corrected/T	73.4	88.6	91.2	93.0	93.8	94.9
ML-fixed USPTO-460k/forward/radius 0 corrected/P	80.7	90.1	92.4	93.7	94.5	95.7
ML-fixed USPTO-460k/forward/radius 0 canonical corrected/T	75.6	88.8	91.3	93.1	94.0	95.0
ML-fixed USPTO-460k/forward/radius 0 canonical corrected/P	81.1	90.3	92.4	93.8	94.8	95.8
ML-learned USPTO-50k/retro/default/T	26.7	43.7	49.5	56.7	62.3	68.1
ML-learned USPTO-50k/retro/default/P	31.1	50.1	56.3	64.4	70.8	77.1
ML-learned USPTO-50k/retro/default canonical/T	27.3	44.2	50.5	57.6	63.1	69.1
ML-learned USPTO-50k/retro/default canonical/P	30.4	49.2	56.2	64.4	70.6	78.0
ML-learned USPTO-50k/retro/default corrected/T	42.3	64.5	72.2	79.3	84.3	88.2
ML-learned USPTO-50k/retro/default corrected/P	42.5	64.8	72.5	79.7	84.6	88.7
ML-learned USPTO-50k/retro/default canonical corrected/T	39.3	61.8	69.7	77.7	82.9	87.7
ML-learned USPTO-50k/retro/default canonical corrected/P	39.4	61.8	69.8	77.9	83.1	87.8
ML-learned USPTO-50k/retro/radius 3/T	3.1	5.0	6.3	7.9	10.0	12.9
ML-learned USPTO-50k/retro/radius 3/P	4.8	7.8	9.2	11.9	14.5	20.2
ML-learned USPTO-50k/retro/radius 3 canonical/T	3.3	5.0	6.5	8.4	10.2	13.4
ML-learned USPTO-50k/retro/radius 3 canonical/P	5.2	7.5	9.4	13.3	15.7	20.9
ML-learned USPTO-50k/retro/radius 3 corrected/T	10.9	17.6	20.5	24.5	29.0	35.9
ML-learned USPTO-50k/retro/radius 3 corrected/P	11.8	18.9	22.3	26.1	30.6	37.1
ML-learned USPTO-50k/retro/radius 3 canonical corrected/T	11.3	18.0	21.6	25.3	29.8	35.9
ML-learned USPTO-50k/retro/radius 3 canonical corrected/P	11.7	18.5	22.3	26.1	30.5	36.6
ML-learned USPTO-50k/retro/radius 2/T	19.6	32.1	37.0	42.2	47.0	52.8
ML-learned USPTO-50k/retro/radius 2/P	22.5	35.6	41.4	47.8	53.4	60.1
ML-learned USPTO-50k/retro/radius 2 canonical/T	20.9	33.5	38.1	43.8	48.8	54.3

Continued on next page

Table S1 – continued from previous page

System	top-1	top-3	top-5	top-10	top-20	top-50
ML-learned USPTO-50k/retro/radius 2 canonical/P	22.6	36.5	41.6	48.5	54.2	61.0
ML-learned USPTO-50k/retro/radius 2 corrected/T	30.4	47.6	54.0	61.7	67.6	73.2
ML-learned USPTO-50k/retro/radius 2 corrected/P	31.0	48.2	54.5	62.3	68.3	74.0
ML-learned USPTO-50k/retro/radius 2 canonical corrected/T	31.2	48.9	54.6	62.0	67.9	73.5
ML-learned USPTO-50k/retro/radius 2 canonical corrected/P	31.6	49.2	54.8	62.3	68.4	74.0
ML-learned USPTO-50k/retro/radius 1/T	37.2	59.7	67.6	75.2	80.9	86.3
ML-learned USPTO-50k/retro/radius 1/P	39.0	61.5	69.3	77.4	82.8	87.9
ML-learned USPTO-50k/retro/radius 1 canonical/T	39.0	61.1	69.1	77.2	82.8	87.8
ML-learned USPTO-50k/retro/radius 1 canonical/P	39.4	61.6	69.8	78.1	83.6	88.5
ML-learned USPTO-50k/retro/radius 1 corrected/T	39.2	61.5	69.8	77.6	82.6	87.6
ML-learned USPTO-50k/retro/radius 1 corrected/P	39.5	62.0	70.2	78.0	82.9	87.8
ML-learned USPTO-50k/retro/radius 1 canonical corrected/T	39.7	62.0	70.0	78.0	83.4	87.7
ML-learned USPTO-50k/retro/radius 1 canonical corrected/P	39.8	62.2	70.1	78.2	83.5	87.9
ML-learned USPTO-50k/retro/radius 0/T	46.1	70.9	79.1	87.7	92.5	95.8
ML-learned USPTO-50k/retro/radius 0/P	47.1	71.8	80.1	88.3	92.9	96.2
ML-learned USPTO-50k/retro/radius 0 canonical/T	47.8	72.9	81.8	89.3	93.6	96.4
ML-learned USPTO-50k/retro/radius 0 canonical/P	47.9	73.0	81.9	89.5	93.9	96.7
ML-learned USPTO-50k/retro/radius 0 corrected/T	46.1	70.9	79.1	87.7	92.5	95.8
ML-learned USPTO-50k/retro/radius 0 corrected/P	47.1	71.8	80.1	88.3	92.9	96.2
ML-learned USPTO-50k/retro/radius 0 canonical corrected/T	47.8	72.9	81.8	89.3	93.6	96.4
ML-learned USPTO-50k/retro/radius 0 canonical corrected/P	47.9	73.0	81.9	89.5	93.9	96.7
ML-learned USPTO-50k/forward/default/T	41.7	54.8	59.4	64.6	68.6	72.7
ML-learned USPTO-50k/forward/default/P	50.9	65.9	70.6	76.7	80.9	85.7
ML-learned USPTO-50k/forward/default canonical/T	43.8	56.8	61.7	67.1	70.9	75.1
ML-learned USPTO-50k/forward/default canonical/P	51.0	67.1	72.4	77.7	82.0	86.1
ML-learned USPTO-50k/forward/default corrected/T	72.4	85.4	88.2	91.0	92.5	93.8
ML-learned USPTO-50k/forward/default corrected/P	73.1	85.6	88.4	91.2	92.7	94.2
ML-learned USPTO-50k/forward/default canonical corrected/T	77.4	88.5	90.7	92.8	94.0	94.9
ML-learned USPTO-50k/forward/default canonical corrected/P	77.2	88.3	90.6	92.6	93.8	94.7
ML-learned USPTO-50k/forward/radius 3/T	4.9	7.4	8.7	10.3	13.1	16.2
ML-learned USPTO-50k/forward/radius 3/P	8.9	12.7	14.0	16.6	20.0	25.2
ML-learned USPTO-50k/forward/radius 3 canonical/T	4.8	8.0	9.2	11.1	13.7	16.5
ML-learned USPTO-50k/forward/radius 3 canonical/P	8.7	13.1	14.6	17.1	20.9	25.6
ML-learned USPTO-50k/forward/radius 3 corrected/T	24.8	36.6	41.1	46.8	51.1	56.3
ML-learned USPTO-50k/forward/radius 3 corrected/P	26.5	38.6	42.9	48.8	53.1	58.4
ML-learned USPTO-50k/forward/radius 3 canonical corrected/T	26.1	38.1	42.3	47.2	51.8	56.9
ML-learned USPTO-50k/forward/radius 3 canonical corrected/P	27.0	39.5	43.8	48.8	53.4	58.6
ML-learned USPTO-50k/forward/radius 2/T	30.9	42.6	47.2	52.7	56.9	62.3

Continued on next page

Table S1 – continued from previous page

System	top-1	top-3	top-5	top-10	top-20	top-50
ML-learned USPTO-50k/forward/radius 2/P	37.3	50.8	55.8	62.1	66.5	71.9
ML-learned USPTO-50k/forward/radius 2 canonical/T	33.7	45.9	50.5	55.6	60.1	65.0
ML-learned USPTO-50k/forward/radius 2 canonical/P	39.2	53.6	58.6	64.2	68.7	73.8
ML-learned USPTO-50k/forward/radius 2 corrected/T	47.3	62.4	67.0	72.0	75.9	79.6
ML-learned USPTO-50k/forward/radius 2 corrected/P	48.8	63.5	68.2	73.4	77.3	81.0
ML-learned USPTO-50k/forward/radius 2 canonical corrected/T	53.8	67.5	71.5	76.1	79.4	82.5
ML-learned USPTO-50k/forward/radius 2 canonical corrected/P	54.9	68.2	72.4	77.0	80.3	83.4
ML-learned USPTO-50k/forward/radius 1/T	66.8	83.0	86.6	90.0	92.1	93.7
ML-learned USPTO-50k/forward/radius 1/P	71.6	84.9	88.3	91.6	93.3	94.8
ML-learned USPTO-50k/forward/radius 1 canonical/T	73.5	86.5	90.0	92.2	93.4	94.7
ML-learned USPTO-50k/forward/radius 1 canonical/P	73.9	86.9	90.3	92.6	93.9	95.3
ML-learned USPTO-50k/forward/radius 1 corrected/T	75.2	88.2	90.7	92.7	94.0	95.0
ML-learned USPTO-50k/forward/radius 1 corrected/P	75.4	88.2	90.8	92.8	94.1	95.3
ML-learned USPTO-50k/forward/radius 1 canonical corrected/T	74.0	87.7	90.4	92.5	94.0	94.9
ML-learned USPTO-50k/forward/radius 1 canonical corrected/P	74.0	87.4	90.3	92.3	93.8	94.8
ML-learned USPTO-50k/forward/radius 0/T	83.7	95.4	96.7	97.6	98.1	98.5
ML-learned USPTO-50k/forward/radius 0/P	85.5	95.2	96.5	97.2	97.6	97.9
ML-learned USPTO-50k/forward/radius 0 canonical/T	85.2	95.7	97.2	98.0	98.3	98.7
ML-learned USPTO-50k/forward/radius 0 canonical/P	84.9	95.2	96.6	97.3	97.7	98.0
ML-learned USPTO-50k/forward/radius 0 corrected/T	83.7	95.4	96.7	97.6	98.1	98.5
ML-learned USPTO-50k/forward/radius 0 corrected/P	85.5	95.2	96.5	97.2	97.6	97.9
ML-learned USPTO-50k/forward/radius 0 canonical corrected/T	85.2	95.7	97.2	98.0	98.3	98.7
ML-learned USPTO-50k/forward/radius 0 canonical corrected/P	84.9	95.2	96.6	97.3	97.7	98.0

S8 Top-N-applicabilities of all systems

Table S2: Top-N-applicabilities of all systems (in percent).

System	top-1	top-5	top-10	top-25	top-50	top-100
Similarity USPTO-50k/retro/default	53.6	43.9	38.4	32.3	28.0	24.1
Similarity USPTO-50k/retro/default canonical	53.6	43.9	38.4	32.3	28.0	24.1
Similarity USPTO-50k/retro/default corrected	60.3	51.7	46.6	40.4	35.8	31.5
Similarity USPTO-50k/retro/default canonical corrected	60.2	51.7	46.6	40.3	35.8	31.5
Similarity USPTO-50k/retro/radius 3	30.7	20.6	16.1	11.6	9.0	6.9
Similarity USPTO-50k/retro/radius 3 canonical	30.7	20.5	16.1	11.5	9.0	6.9
Similarity USPTO-50k/retro/radius 3 corrected	39.1	28.9	23.6	17.9	14.4	11.6
Similarity USPTO-50k/retro/radius 3 canonical corrected	39.2	29.0	23.8	18.1	14.7	11.7
Similarity USPTO-50k/retro/radius 2	45.6	35.2	29.7	23.7	19.8	16.4
Similarity USPTO-50k/retro/radius 2 canonical	45.6	35.1	29.7	23.7	19.8	16.4
Similarity USPTO-50k/retro/radius 2 corrected	49.4	39.6	34.1	27.8	23.6	19.9
Similarity USPTO-50k/retro/radius 2 canonical corrected	49.3	39.6	34.1	27.9	23.7	19.9
Similarity USPTO-50k/retro/radius 1	60.8	52.3	47.3	40.9	36.4	32.1
Similarity USPTO-50k/retro/radius 1 canonical	60.8	52.3	47.3	40.9	36.4	32.1
Similarity USPTO-50k/retro/radius 1 corrected	60.7	52.2	47.2	40.9	36.4	32.1
Similarity USPTO-50k/retro/radius 1 canonical corrected	60.6	52.2	47.2	40.9	36.4	32.1
Similarity USPTO-50k/retro/radius 0	72.8	66.7	63.1	58.4	54.9	51.3
Similarity USPTO-50k/retro/radius 0 canonical	72.8	66.7	63.1	58.4	54.9	51.3
Similarity USPTO-50k/retro/radius 0 corrected	72.8	66.7	63.1	58.4	54.9	51.3
Similarity USPTO-50k/retro/radius 0 canonical corrected	72.8	66.7	63.1	58.4	54.9	51.3
Similarity USPTO-50k/forward/default	46.4	35.2	30.4	24.7	21.4	18.6
Similarity USPTO-50k/forward/default canonical	46.4	35.2	30.4	24.7	21.4	18.6
Similarity USPTO-50k/forward/default corrected	51.1	40.5	35.7	30.0	26.4	23.1
Similarity USPTO-50k/forward/default canonical corrected	51.1	40.5	35.7	30.0	26.4	23.1
Similarity USPTO-50k/forward/radius 3	27.9	17.9	13.9	10.2	8.1	6.5
Similarity USPTO-50k/forward/radius 3 canonical	27.9	17.9	13.9	10.1	8.1	6.5
Similarity USPTO-50k/forward/radius 3 corrected	35.1	24.4	19.8	15.0	12.3	10.1
Similarity USPTO-50k/forward/radius 3 canonical corrected	35.2	24.6	20.1	15.3	12.6	10.3
Similarity USPTO-50k/forward/radius 2	40.2	29.0	24.5	19.3	16.3	13.8
Similarity USPTO-50k/forward/radius 2 canonical	40.2	29.0	24.5	19.3	16.3	13.7
Similarity USPTO-50k/forward/radius 2 corrected	43.5	32.3	27.6	22.2	19.0	16.2
Similarity USPTO-50k/forward/radius 2 canonical corrected	43.4	32.4	27.7	22.3	19.1	16.3
Similarity USPTO-50k/forward/radius 1	51.5	41.0	36.3	30.6	27.0	23.7
Similarity USPTO-50k/forward/radius 1 canonical	51.5	41.0	36.3	30.6	27.0	23.7
Similarity USPTO-50k/forward/radius 1 corrected	51.5	41.1	36.4	30.6	27.0	23.7

Continued on next page

Table S2 – continued from previous page

System	top-1	top-3	top-5	top-10	top-20	top-50
Similarity USPTO-50k/forward/radius 1 canonical corrected	51.5	41.1	36.4	30.6	27.0	23.7
Similarity USPTO-50k/forward/radius 0	58.9	49.7	45.4	40.2	36.8	33.5
Similarity USPTO-50k/forward/radius 0 canonical	58.9	49.7	45.4	40.2	36.8	33.5
Similarity USPTO-50k/forward/radius 0 corrected	58.9	49.7	45.4	40.2	36.8	33.5
Similarity USPTO-50k/forward/radius 0 canonical corrected	58.9	49.7	45.4	40.2	36.8	33.5
Similarity USPTO-460k/retro/default	68.3	58.8	53.3	45.7	39.8	34.3
Similarity USPTO-460k/retro/default canonical	68.3	58.8	53.3	45.7	39.8	34.3
Similarity USPTO-460k/retro/default corrected	72.6	64.0	58.9	51.8	46.3	41.0
Similarity USPTO-460k/retro/default canonical corrected	72.6	64.0	58.9	51.8	46.3	41.0
Similarity USPTO-460k/retro/radius 3	49.9	38.5	32.5	24.8	19.7	15.3
Similarity USPTO-460k/retro/radius 3 canonical	49.9	38.5	32.5	24.8	19.7	15.3
Similarity USPTO-460k/retro/radius 3 corrected	57.5	46.6	40.7	32.7	27.1	22.1
Similarity USPTO-460k/retro/radius 3 canonical corrected	57.8	46.7	40.9	32.9	27.2	22.2
Similarity USPTO-460k/retro/radius 2	62.7	51.6	45.7	37.8	32.1	26.7
Similarity USPTO-460k/retro/radius 2 canonical	62.7	51.6	45.7	37.8	32.1	26.7
Similarity USPTO-460k/retro/radius 2 corrected	65.7	55.3	49.6	42.0	36.2	30.7
Similarity USPTO-460k/retro/radius 2 canonical corrected	65.8	55.2	49.6	41.9	36.2	30.7
Similarity USPTO-460k/retro/radius 1	73.3	64.9	59.7	52.6	47.1	41.7
Similarity USPTO-460k/retro/radius 1 canonical	73.3	64.9	59.7	52.6	47.1	41.7
Similarity USPTO-460k/retro/radius 1 corrected	73.4	64.9	59.8	52.7	47.1	41.8
Similarity USPTO-460k/retro/radius 1 canonical corrected	73.4	64.9	59.8	52.7	47.1	41.7
Similarity USPTO-460k/retro/radius 0	80.4	73.7	69.8	64.5	60.1	55.9
Similarity USPTO-460k/retro/radius 0 canonical	80.4	73.7	69.8	64.5	60.1	55.9
Similarity USPTO-460k/retro/radius 0 corrected	80.4	73.7	69.8	64.5	60.1	55.9
Similarity USPTO-460k/retro/radius 0 canonical corrected	80.4	73.7	69.8	64.5	60.1	55.9
Similarity USPTO-460k/forward/default	60.7	48.6	42.6	34.6	29.1	24.3
Similarity USPTO-460k/forward/default canonical	60.7	48.6	42.6	34.6	29.1	24.3
Similarity USPTO-460k/forward/default corrected	64.3	52.7	46.9	39.0	33.5	28.5
Similarity USPTO-460k/forward/default canonical corrected	64.3	52.7	46.9	39.0	33.5	28.5
Similarity USPTO-460k/forward/radius 3	45.5	32.5	26.9	19.8	15.4	11.9
Similarity USPTO-460k/forward/radius 3 canonical	45.5	32.5	26.8	19.8	15.4	11.9
Similarity USPTO-460k/forward/radius 3 corrected	51.9	39.3	33.5	25.9	20.9	16.7
Similarity USPTO-460k/forward/radius 3 canonical corrected	51.9	39.4	33.6	26.0	21.1	16.9
Similarity USPTO-460k/forward/radius 2	55.8	43.2	37.2	29.4	24.2	19.7
Similarity USPTO-460k/forward/radius 2 canonical	55.8	43.2	37.2	29.4	24.2	19.7
Similarity USPTO-460k/forward/radius 2 corrected	58.3	46.1	40.1	32.3	26.9	22.2
Similarity USPTO-460k/forward/radius 2 canonical corrected	58.4	46.0	40.1	32.3	27.0	22.3
Similarity USPTO-460k/forward/radius 1	64.5	52.9	47.2	39.4	33.8	28.9

Continued on next page

Table S2 – continued from previous page

System	top-1	top-3	top-5	top-10	top-20	top-50
Similarity USPTO-460k/forward/radius 1 canonical	64.5	52.9	47.2	39.3	33.8	28.9
Similarity USPTO-460k/forward/radius 1 corrected	64.6	53.0	47.2	39.4	33.9	28.9
Similarity USPTO-460k/forward/radius 1 canonical corrected	64.6	53.0	47.2	39.4	33.9	28.9
Similarity USPTO-460k/forward/radius 0	68.9	58.8	53.8	46.7	41.7	37.1
Similarity USPTO-460k/forward/radius 0 canonical	68.9	58.8	53.8	46.7	41.7	37.1
Similarity USPTO-460k/forward/radius 0 corrected	68.9	58.8	53.8	46.7	41.7	37.1
Similarity USPTO-460k/forward/radius 0 canonical corrected	68.9	58.8	53.8	46.7	41.7	37.1
ML-fixed USPTO-50k/retro/default	85.1	64.4	50.9	33.8	23.7	16.3
ML-fixed USPTO-50k/retro/default canonical	86.3	65.2	50.8	32.9	22.7	15.5
ML-fixed USPTO-50k/retro/default corrected	93.5	76.8	63.7	44.0	31.3	21.2
ML-fixed USPTO-50k/retro/default canonical corrected	93.8	77.8	65.0	45.2	31.8	21.3
ML-fixed USPTO-50k/retro/radius 3	42.7	24.5	16.7	9.5	6.1	3.9
ML-fixed USPTO-50k/retro/radius 3 canonical	42.7	24.4	16.6	9.4	6.0	3.8
ML-fixed USPTO-50k/retro/radius 3 corrected	60.0	38.0	27.5	16.7	10.9	6.9
ML-fixed USPTO-50k/retro/radius 3 canonical corrected	59.8	37.6	26.8	15.9	10.5	6.7
ML-fixed USPTO-50k/retro/radius 2	74.6	51.2	37.5	22.1	14.3	9.1
ML-fixed USPTO-50k/retro/radius 2 canonical	75.6	50.2	36.5	21.7	14.0	9.0
ML-fixed USPTO-50k/retro/radius 2 corrected	80.5	56.6	42.7	26.1	17.3	11.4
ML-fixed USPTO-50k/retro/radius 2 canonical corrected	80.7	56.0	41.7	25.6	17.0	10.9
ML-fixed USPTO-50k/retro/radius 1	93.5	78.4	66.3	47.2	34.0	23.6
ML-fixed USPTO-50k/retro/radius 1 canonical	94.2	78.5	65.7	45.4	32.1	21.8
ML-fixed USPTO-50k/retro/radius 1 corrected	93.4	77.1	64.0	44.4	31.6	21.8
ML-fixed USPTO-50k/retro/radius 1 canonical corrected	94.0	77.9	64.9	44.9	31.8	21.8
ML-fixed USPTO-50k/retro/radius 0	97.9	90.0	81.6	66.7	53.7	40.9
ML-fixed USPTO-50k/retro/radius 0 canonical	97.8	90.1	81.6	66.0	52.2	39.3
ML-fixed USPTO-50k/retro/radius 0 corrected	97.9	90.0	81.6	66.7	53.7	40.9
ML-fixed USPTO-50k/retro/radius 0 canonical corrected	97.8	90.1	81.6	66.0	52.2	39.3
ML-fixed USPTO-50k/forward/default	81.9	40.8	27.3	15.4	9.7	6.0
ML-fixed USPTO-50k/forward/default canonical	82.2	39.2	25.9	14.5	9.2	5.7
ML-fixed USPTO-50k/forward/default corrected	91.3	42.9	28.5	15.8	9.7	5.8
ML-fixed USPTO-50k/forward/default canonical corrected	90.4	42.1	27.7	15.4	9.5	5.8
ML-fixed USPTO-50k/forward/radius 3	46.7	20.3	13.2	7.1	4.3	2.6
ML-fixed USPTO-50k/forward/radius 3 canonical	46.9	19.9	12.8	6.8	4.2	2.5
ML-fixed USPTO-50k/forward/radius 3 corrected	57.1	24.0	15.1	7.8	4.7	2.7
ML-fixed USPTO-50k/forward/radius 3 canonical corrected	58.3	23.6	14.7	7.7	4.6	2.7
ML-fixed USPTO-50k/forward/radius 2	69.8	30.9	19.7	10.4	6.3	3.7
ML-fixed USPTO-50k/forward/radius 2 canonical	70.0	28.8	18.5	9.8	5.9	3.5
ML-fixed USPTO-50k/forward/radius 2 corrected	77.3	32.8	21.1	11.3	6.9	4.0

Continued on next page

Table S2 – continued from previous page

System	top-1	top-3	top-5	top-10	top-20	top-50
ML-fixed USPTO-50k/forward/radius 2 canonical corrected	76.1	31.0	19.7	10.5	6.4	3.8
ML-fixed USPTO-50k/forward/radius 1	90.2	44.4	29.5	16.5	10.3	6.3
ML-fixed USPTO-50k/forward/radius 1 canonical	91.5	43.9	29.6	16.6	10.3	6.2
ML-fixed USPTO-50k/forward/radius 1 corrected	91.1	42.4	28.6	16.2	10.1	6.2
ML-fixed USPTO-50k/forward/radius 1 canonical corrected	91.3	42.7	28.1	15.8	10.0	6.1
ML-fixed USPTO-50k/forward/radius 0	97.1	59.7	44.5	28.3	19.3	12.7
ML-fixed USPTO-50k/forward/radius 0 canonical	97.1	57.6	41.8	25.5	16.9	10.8
ML-fixed USPTO-50k/forward/radius 0 corrected	97.1	59.7	44.5	28.3	19.3	12.7
ML-fixed USPTO-50k/forward/radius 0 canonical corrected	97.1	57.6	41.8	25.5	16.9	10.8
ML-fixed USPTO-460k/retro/default	91.4	76.4	66.3	50.6	38.6	28.0
ML-fixed USPTO-460k/retro/default canonical	91.9	75.9	65.8	50.1	38.2	27.9
ML-fixed USPTO-460k/retro/default corrected	96.0	84.6	75.6	60.5	47.7	35.7
ML-fixed USPTO-460k/retro/default canonical corrected	95.9	84.2	75.1	59.9	47.4	35.4
ML-fixed USPTO-460k/retro/radius 3	64.0	42.1	32.2	20.4	13.7	9.0
ML-fixed USPTO-460k/retro/radius 3 canonical	66.0	42.6	31.7	19.7	13.2	8.6
ML-fixed USPTO-460k/retro/radius 3 corrected	78.2	55.4	44.4	30.5	21.9	15.1
ML-fixed USPTO-460k/retro/radius 3 canonical corrected	78.8	55.9	45.0	30.9	22.0	15.1
ML-fixed USPTO-460k/retro/radius 2	85.8	66.5	54.9	38.6	27.7	18.9
ML-fixed USPTO-460k/retro/radius 2 canonical	86.3	65.8	53.7	37.5	26.7	18.4
ML-fixed USPTO-460k/retro/radius 2 corrected	89.0	70.9	59.6	43.5	32.2	22.9
ML-fixed USPTO-460k/retro/radius 2 canonical corrected	89.4	71.0	59.8	43.9	32.9	23.4
ML-fixed USPTO-460k/retro/radius 1	95.8	85.6	77.2	62.8	50.1	37.9
ML-fixed USPTO-460k/retro/radius 1 canonical	96.1	84.9	75.9	60.9	48.4	36.3
ML-fixed USPTO-460k/retro/radius 1 corrected	96.0	84.7	75.9	60.7	48.1	36.1
ML-fixed USPTO-460k/retro/radius 1 canonical corrected	95.9	84.9	75.6	60.6	48.2	36.2
ML-fixed USPTO-460k/retro/radius 0	98.8	93.9	88.9	78.9	69.0	58.1
ML-fixed USPTO-460k/retro/radius 0 canonical	98.8	93.9	89.0	78.7	68.5	57.5
ML-fixed USPTO-460k/retro/radius 0 corrected	98.8	93.9	88.9	78.9	69.0	58.1
ML-fixed USPTO-460k/retro/radius 0 canonical corrected	98.8	93.9	89.0	78.7	68.5	57.5
ML-fixed USPTO-460k/forward/default	86.8	49.9	35.1	21.3	14.3	9.5
ML-fixed USPTO-460k/forward/default canonical	87.1	48.6	34.0	20.6	13.8	9.2
ML-fixed USPTO-460k/forward/default corrected	91.4	52.8	37.5	23.0	15.7	10.5
ML-fixed USPTO-460k/forward/default canonical corrected	92.2	53.1	37.9	23.4	16.0	10.6
ML-fixed USPTO-460k/forward/radius 3	58.1	27.9	18.4	10.1	6.3	3.9
ML-fixed USPTO-460k/forward/radius 3 canonical	58.5	26.1	17.0	9.2	5.7	3.5
ML-fixed USPTO-460k/forward/radius 3 corrected	71.3	33.0	21.8	12.2	7.8	4.8
ML-fixed USPTO-460k/forward/radius 3 canonical corrected	71.5	32.9	21.4	12.0	7.6	4.8
ML-fixed USPTO-460k/forward/radius 2	79.1	41.0	27.2	15.3	9.8	6.2

Continued on next page

Table S2 – continued from previous page

System	top-1	top-3	top-5	top-10	top-20	top-50
ML-fixed USPTO-460k/forward/radius 2 canonical	79.8	38.6	25.7	14.5	9.3	5.9
ML-fixed USPTO-460k/forward/radius 2 corrected	83.9	41.5	27.9	16.1	10.6	6.8
ML-fixed USPTO-460k/forward/radius 2 canonical corrected	84.0	41.6	27.8	16.0	10.6	6.8
ML-fixed USPTO-460k/forward/radius 1	91.9	55.4	39.6	24.5	16.9	11.4
ML-fixed USPTO-460k/forward/radius 1 canonical	92.5	53.6	38.2	23.8	16.5	11.2
ML-fixed USPTO-460k/forward/radius 1 corrected	92.5	52.9	37.5	23.3	16.2	11.1
ML-fixed USPTO-460k/forward/radius 1 canonical corrected	92.3	53.6	38.0	23.7	16.4	11.1
ML-fixed USPTO-460k/forward/radius 0	96.3	70.6	56.9	40.8	30.6	22.4
ML-fixed USPTO-460k/forward/radius 0 canonical	96.7	69.6	56.0	39.9	29.7	21.7
ML-fixed USPTO-460k/forward/radius 0 corrected	96.3	70.6	56.9	40.8	30.6	22.4
ML-fixed USPTO-460k/forward/radius 0 canonical corrected	96.7	69.6	56.0	39.9	29.7	21.7
ML-learned USPTO-50k/retro/default	69.7	50.2	39.1	26.4	18.8	13.3
ML-learned USPTO-50k/retro/default canonical	68.2	48.7	37.5	24.9	17.7	12.4
ML-learned USPTO-50k/retro/default corrected	89.6	70.5	56.8	38.4	27.4	18.9
ML-learned USPTO-50k/retro/default canonical corrected	87.6	68.7	55.5	37.5	26.7	18.4
ML-learned USPTO-50k/retro/radius 3	8.4	5.6	4.8	3.2	2.4	1.7
ML-learned USPTO-50k/retro/radius 3 canonical	9.3	5.5	5.3	3.3	2.3	1.5
ML-learned USPTO-50k/retro/radius 3 corrected	28.5	19.5	14.4	8.8	6.0	4.1
ML-learned USPTO-50k/retro/radius 3 canonical corrected	29.7	20.0	14.5	8.6	5.9	4.0
ML-learned USPTO-50k/retro/radius 2	55.7	39.1	28.2	17.1	11.5	7.6
ML-learned USPTO-50k/retro/radius 2 canonical	55.3	38.0	27.4	16.6	11.1	7.4
ML-learned USPTO-50k/retro/radius 2 corrected	68.3	46.8	35.1	22.2	15.0	10.0
ML-learned USPTO-50k/retro/radius 2 canonical corrected	68.1	46.0	34.2	21.4	14.5	9.6
ML-learned USPTO-50k/retro/radius 1	86.7	69.1	56.7	39.6	28.8	20.4
ML-learned USPTO-50k/retro/radius 1 canonical	87.8	69.5	56.3	38.5	27.6	19.3
ML-learned USPTO-50k/retro/radius 1 corrected	87.7	69.4	56.2	38.4	27.6	19.3
ML-learned USPTO-50k/retro/radius 1 canonical corrected	87.7	70.5	57.3	39.3	28.1	19.5
ML-learned USPTO-50k/retro/radius 0	97.8	91.2	83.3	67.2	53.0	39.2
ML-learned USPTO-50k/retro/radius 0 canonical	98.1	92.1	85.4	69.2	53.8	39.0
ML-learned USPTO-50k/retro/radius 0 corrected	97.8	91.2	83.3	67.2	53.0	39.2
ML-learned USPTO-50k/retro/radius 0 canonical corrected	98.1	92.1	85.4	69.2	53.8	39.0
ML-learned USPTO-50k/forward/default	62.4	28.0	19.0	11.3	7.6	5.0
ML-learned USPTO-50k/forward/default canonical	61.8	26.7	18.0	10.6	7.1	4.7
ML-learned USPTO-50k/forward/default corrected	84.7	34.4	22.6	13.3	8.8	5.6
ML-learned USPTO-50k/forward/default canonical corrected	88.3	38.1	25.8	14.9	9.7	5.9
ML-learned USPTO-50k/forward/radius 3	23.5	11.1	7.4	4.2	2.6	1.6
ML-learned USPTO-50k/forward/radius 3 canonical	19.4	9.6	6.4	3.7	2.3	1.4
ML-learned USPTO-50k/forward/radius 3 corrected	33.8	15.1	9.9	5.6	3.5	2.2

Continued on next page

Table S2 – continued from previous page

System	top-1	top-3	top-5	top-10	top-20	top-50
ML-learned USPTO-50k/forward/radius 3 canonical corrected	34.4	15.0	9.7	5.4	3.4	2.1
ML-learned USPTO-50k/forward/radius 2	48.3	22.0	14.5	8.1	5.1	3.2
ML-learned USPTO-50k/forward/radius 2 canonical	48.6	20.8	13.6	7.5	4.8	3.0
ML-learned USPTO-50k/forward/radius 2 corrected	58.8	23.3	14.9	8.4	5.4	3.4
ML-learned USPTO-50k/forward/radius 2 canonical corrected	64.1	23.6	14.9	8.3	5.4	3.4
ML-learned USPTO-50k/forward/radius 1	83.0	36.9	24.7	14.5	9.7	6.3
ML-learned USPTO-50k/forward/radius 1 canonical	86.0	37.3	25.4	14.9	9.9	6.2
ML-learned USPTO-50k/forward/radius 1 corrected	87.2	37.5	25.3	15.0	9.8	6.2
ML-learned USPTO-50k/forward/radius 1 canonical corrected	85.9	36.7	24.7	14.6	9.6	6.1
ML-learned USPTO-50k/forward/radius 0	96.8	55.2	40.0	25.8	17.8	11.9
ML-learned USPTO-50k/forward/radius 0 canonical	97.0	54.5	40.0	25.0	16.8	10.8
ML-learned USPTO-50k/forward/radius 0 corrected	96.8	55.2	40.0	25.8	17.8	11.9
ML-learned USPTO-50k/forward/radius 0 canonical corrected	97.0	54.5	40.0	25.0	16.8	10.8

References

(1) Coley, C. W.; Rogers, L.; Green, W. H.; Jensen, K. F. Computer-assisted Retrosynthesis Based on Molecular Similarity. *ACS Cent. Sci.* **2017**, *3*, 1237–1245.

(2) Landrum, G. RDKit: Open-source Cheminformatics. 2006; `https://www.rdkit.org/`, https://www.rdkit.org/.

(3) Fortunato, M. E.; Coley, C. W.; Barnes, B. C.; Jensen, K. F. Data Augmentation and Pretraining for Template-based Retrosynthetic Prediction in Computer-aided Synthesis Planning. *J. Chem. Inf. Model.* **2020**, *60*, 3398–3407.

(4) Yang, K.; Swanson, K.; Jin, W.; Coley, C.; Eiden, P.; Gao, H.; Guzman-Perez, A.; Hopper, T.; Kelley, B.; Mathea, M.; Palmer, A.; Settels, V.; Jaakkola, T.; Jensen, K.; Barzilay, R. Analyzing Learned Molecular Representations for Property Prediction. *J. Chem. Inf. Model.* **2019**, *59*, 3370–3388.
